# Sex Discrepancy Observed for Gestational Metabolic Syndrome Parameters and Polygenic Risk Associated With Preschoolers’ BMI Growth Trajectory: The Ma’anshan Birth Cohort Study

**DOI:** 10.3389/fendo.2022.857711

**Published:** 2022-07-01

**Authors:** Bei-bei Zhu, Hui Gao, Meng-long Geng, Xiulong Wu, Juan Tong, Fen Deng, Si-ying Zhang, Li-hong Wu, Kun Huang, Xiao-yan Wu, Hong Gan, Peng Zhu, Fang-biao Tao

**Affiliations:** ^1^ Department of Maternal, Child and Adolescent Health, School of Public Health, Anhui Medical University, Hefei, China; ^2^ Key Laboratory of Population Health Across Life Cycle (Anhui Medical University), Ministry of Education of the People’s Republic of China, Hefei, China; ^3^ Department of Pediatrics, The First Affiliated Hospital of Anhui Medical University, Hefei, China; ^4^ Key Laboratory of Study on Abnormal Gametes and Reproductive Tract, (Anhui Medical University), National Health Commission of the People’s Republic China, Hefei, China; ^5^ Anhui Provincial Key Laboratory of Population Health and Aristogenics, Anhui Medical University, Hefei, China

**Keywords:** gestational metabolic syndrome, polygenic risk, body mass index trajectory, interaction, sex discrepancy, prospective birth cohort study

## Abstract

**Background:**

Few studies have investigated the associations of childhood growth trajectories with the prenatal metabolic risks of mothers and their interaction with children’s genetic susceptibility.

**Objective:**

To investigate the effects of gestational metabolic syndrome (GMS) risks and children’s polygenic risk scores (PRSs), and their interaction effect on the BMI trajectory and obesity risk of offspring from birth to 6 years of age.

**Methods:**

A total of 2,603 mother-child pairs were recruited from the Ma’anshan birth cohort (Anhui Province of China) study. Data on maternal prepregnancy obesity, gestational weight gain (GWG), gestational diabetes mellitus (GDM), and hypertensive disorders of pregnancy (HDP) were used to evaluate maternal GMS risk. In addition, 1,482 cord blood samples were used to genotype 11 candidate single-nucleotide polymorphisms (SNPs) to calculate children’s PRSs. The latent class growth model using the longitudinal BMI-for-age z scores (BMIz) was applied to validly capture the BMIz growth trajectory.

**Results:**

Maternal GMS status was associated with higher BMIz scores and with an increased risk of overweight/obesity. Positive relationships were revealed between PRS and the risk of overweight/obesity among girls. Additionally, maternal GMS significantly interacted with the child’s PRS on BMIz scores and the risk of overweight/obesity among girls. Hierarchical BMI trajectory graphs by different exposure groups showed consistent findings, and both boys’ and girls’ BMIz trajectories were divided into three groups. Among girls, the higher the GMS risk or PRS they had, the higher the probability of being in the high BMIz trajectory group.

**Conclusions:**

Maternal GMS status increased BMIz scores and the risk of obesity in both boys and girls and elevated the child’s BMI trajectory from birth to 6 years of age among girls. PRSs were significantly associated with children’s BMI trajectory and the risk of obesity and modified the associations between maternal GMS status and obesity biomarkers only among girls. Thus, regarding childhood obesity, steps should be taken to decrease maternal metabolic risks before and during pregnancy, and sex discrepancies should be noted to identify high-risk populations after birth to hierarchically manage them.

## Introduction

Childhood obesity is emerging as a public health concern globally (1). In 1975, approximately 4% of children and adolescents aged 5–19 years were overweight and obese; however, the prevalence rose dramatically to over 18% in 2016 (yielding over 340 million) (2). In China, approximately one in seven to eight preschool children are overweight or obese when applying Chinese body mass index (BMI) criteria (3). Obesity begins in childhood and continues over time and has been proven to pose higher risks of diabetes, premature death, and disability in adulthood, so the prevention of childhood obesity creates a critical opportunity for the prevention of chronic diseases in adulthood (4).

BMI is often used as an indicator for the measurement of obesity. Compared with the absolute level of BMI, the longitudinal BMI trajectory, integrating multilevel information such as birth size, BMI gain velocity, and BMI peaks, might be better in predicting asthma, obesity, diabetes, and cardiovascular risk (5, 6). Thus, to comprehensively formulate preventive strategies, longitudinal studies of BMI could be an effective tool.

Identifying risk factors related to childhood obesity is the first step in prevention and reducing its long-term health outcomes. Accumulated evidence has pointed out the importance of intrauterine events in the origins of childhood obesity, and maternal metabolic conditions during pregnancy are one of them (7, 8). A large body of literature has indicated that prenatal metabolic-related factors could increase the risk for childhood obesity (9, 10). However, previous studies have focused on a single metabolic factor; few studies have considered them systematically, and metabolic syndrome (MS) has emerged as a concept that could fill this gap.

To date, there are no obligatory components to define MS (11) but rather a collection of risk factors that encompass metabolic, vascular, and inflammatory aspects (12), often including obesity, hypertension, glucose, intolerance, and dyslipidemia (13). Insulin resistance and hyperinsulinemia may be the basic common ground for MS (14). Pregnant women may develop MS before or during the pregnancy, referred to as gestational MS (GMS). Depicting the relationship between GMS and child BMI trajectory could provide valuable information for developing strategies for the early prevention of childhood obesity. However, few studies to date have focused on this direction (15, 16), leaving a huge research gap to explore.

Environmental factors can be substantially modified by genetic factors, especially for obesity, because previous family and twin studies have indicated that 40%~70% of the interindividual variation in obesity can be explained by genetic components (17). Polygenic risk scores (PRSs) are to summarize the impacts of many genetic variants across the human genome into a single score. Recently, the PRS has been revealed to exhibit a predictive value for multiple common diseases (18), including obesity (19). However, to our knowledge, no study has investigated to what extent GMS parameters can be modified by children’s polygenic risk of childhood obesity.

Therefore, we conducted a prospective study nested on the Ma’anshan birth cohort (MABC). This study aimed to (1) estimate the effects of GMS components, including prepregnancy overweight/obesity, excessive gestational weight gain (GWG), gestational diabetes mellitus (GDM), and hypertensive disorders of pregnancy (HDP), on the BMI trajectory in children up to age 6; (2) explore the associations between PRSs and children’s BMI trajectory; (3) evaluate the interaction between the PRSs and the GMS on children’s BMI growth trajectory; and (4) reveal a sex discrepancy in these potential relationships.

## Materials and Methods

### The MABC Study

The MABC study in Anhui Province in China aimed to investigate the impacts of environmental chemicals on maternal and child health. From May 2013 to September 2014, this study recruited 3,474 eligible pregnant women when they attended their first antenatal care visit at the Ma’anshan Maternal and Child Health Centre. The inclusion criteria were as follows: at least 18 years of age, gestational age of fewer than 14 weeks, not suffering from serious neuropsychiatric disorders, and not planning to move. Women completed questionnaires and provided biological samples at enrollment and were followed up at each trimester of pregnancy. Up to 19 postnatal visits were conducted for mothers and their 3,273 singleton live births. Detailed procedures of the enrollment, follow-up, and biological sample collection of mother-child pairs are described elsewhere (20). The study protocol was approved by the Ethics and Research Committees of Anhui Medical University (Ref. No. 20131195). Written informed consent was obtained from children’s guardians before their recruitment for the MABC study.

### Study Population

Data from the MABC study were used for *post hoc* analyses in this study. A total of 3,273 live singleton neonates were evaluated for height and body weight from birth to 6 years of age. Physical examinations were performed every 3 months from 0 to 1 year of age, every 6 months from 1–3 years of age, and every year from 3–6 years of age. A total of 55 children were excluded due to missing every visit during childhood. To validly capture BMI growth trajectory (the most interesting outcome of this study), a total of 600 children were excluded because of missing physical examinations for three visits in a row during any period of childhood. Furthermore, four women suffering from prepregnancy chronic hypertension, nine women suffering from prepregnancy diabetes mellitus, and two women having both statuses prior to pregnancy were excluded. Finally, 2,603 mother-child pairs were included in this study. Maternal and child characteristics were obtained from questionnaires or abstracted from medical records by uniformly trained staff.

### GMS Component Measurements

Prepregnancy body weight was reported by pregnant women themselves when enrolled. Prepregnancy BMI was defined as body weight (kg) divided by the square of body height (m^2^). Prepregnancy BMI was divided into three groups: (1) underweight (<18.5 kg/m^2^), (2) normal weight (18.5–23.9 kg/m^2^), and (3) overweight (24.0–27.9 kg/m^2^)/obese (≥28.0 kg/m^2^), according to the standard of the Working Group on Obesity in China (21). Maternal weight during the first and second trimesters and before delivery was measured by trained staff. GWG was divided into three classes: (1) insufficient GWG, (2) adequate GWG, and (3) excessive GWG, according to recommendations for total weight gain and the velocity of weight gain during pregnancy by the updated Institute of Medicine guidelines in 2009 (**Supplemental Material, Table S1**) (22). In brief, the appropriate weight gain during the first trimester of pregnancy was 0.5–2.0 kg, regardless of prepregnancy BMI; the appropriate weight gain during later pregnancy from 13 weeks of gestation to within 2 weeks before delivery was calculated, according to the recommendations in **Table S1**: 0.45–0.60 kg/week for the underweight group, 0.36–0.45 kg/week for the normal group, 0.23–0.32 kg/week for the overweight group and 0.18–0.27 kg/week for the obese group. According to data on maternal body weight measured during pregnancy and corresponding gestational weeks, women were divided into three categories (**Supplemental Material, Table S1**).

The GDM and HDP statuses (yes or no) were extracted from the medical records. Women received a 2-hour oral glucose tolerance test with a 75-g glucose load between gestational weeks 24 and 28. The diagnosis of GDM was made if any of the following criteria from the 2014 “Guidelines for the Diagnosis and Treatment of Gestational Diabetes” were met: fasting glucose ≥ 5.1 mmol/L, 1-hour glucose ≥ 10.0 mmol/L, or 2-hour glucose ≥ 8.5 mmol/L. HDP was women self-reported by women who had doctor-diagnosed hypertension or received some hypertensive treatment on the questionnaire (23), including gestational hypertension [systolic pressure (SP) ≥ 140 mmHg or diastolic pressure (DP) ≥ 90 mmHg after gestation] and preeclampsia (SP ≥ 140 mmHg or DP ≥ 90 mmHg with proteinuria ≥ 0.3 g in 24 hours or random urine protein ≥ + after gestational 20 weeks).

Due to the lack of data on blood lipids (e.g., high-density lipoprotein cholesterol and triglycerides), the diagnosis of GMS was limited. Therefore, GMS was divided into four categories according to whether there was overweight, excessive GWG, GDM, or HDP: (1) no risk, none of the above four statuses; (2) low risk, only one of the four statuses; (3) medium risk, two of the four statuses; and (4) high risk, three of the four statuses and above.

### Single-Nucleotide Polymorphism Selection and Genotyping

Fifteen single-nucleotide polymorphisms (SNPs) that reached genome-wide significance for childhood BMI from a joint analysis of 20 genome-wide association studies (GWASs) with a total of 35,668 children were selected (24). Two SNPs (rs7550711 and rs13387838) were excluded because the minor allele frequencies were below 5%, and another two SNPs (rs13253111 and rs4854349) were excluded due to genotyping failure.

DNA was extracted from 1,482 cord blood samples using Roche MagNA Pure 24. The characteristics of the populations with and without gene data are shown in the **Supplemental Material, Table S2**. Genotyping of candidate SNPs was detected by an improved multiple ligase detection reaction method (the Center for Human Genetics Research, Shanghai Genesky Biotechnology Company). To control quality, 5% duplicate samples were independently examined in a blinded way. SNPs had to agree with the Hardy–Weinberg equilibrium, otherwise, would be removed. The call rate of these SNPs was over 99%.

### PRS Calculation

For SNPs, based on the number of risk alleles, three genotypes of each SNP were assigned as 0, 1, or 2 in SPSS 16.0. The sum of the numbers of all BMI risk alleles was used to calculate the PRS. In the present study, the range of PRS was from 3 to 15. A higher PRS indicated a higher genetic predisposition to obesity (24–26). PRSs were then classified into two categories: low-risk (<P_75_) and high-risk (≥P_75_) groups, according to the 75th percentiles.

### Main Outcome Measurement

From birth to 6 years of age, the body weight and height of the children were measured by a physician at each visit. Child BMI (kg/m^2^) was also calculated as weight divided by the square of height. Using SPSS software to run the document http://www.who.int/childgrowth/standards/en/, BMI-for-age z scores (BMIz) were attained according to the WHO Child Growth Standards (27). At each visit, a BMIz ≥ P_85_ was defined as overweight/obesity (28).

The latent class growth model using longitudinal BMIz data was applied to validly capture the BMIz growth trajectory in Mplus 7.0 software, which was also used in our previous study (29). Parameter estimation and model fitting of the latent BMIz growth trajectory were performed by the Lo–Mendell–Rubin maximum likelihood method (30). According to the Bayesian information criterion (BIC) in the group-based trajectory modeling, the optimal number of trajectory groups with different trajectory patterns and the appropriate functions to describe the distinct trajectory patterns were obtained (30). Considering the discrepancy in BMI trajectory between boys (N = 1 276) and girls (N = 1 327), the latent class growth models were performed stratified by sex. According to the statistical analysis results (**Supplemental Material, Figure S1**) and professional knowledge, the growth trajectories were categorized into three patterns for both boys and girls, named “low BMI trajectory” (384 females and 313 males, 26.78%), “medium BMI trajectory” (697 females and 709 males, 54.01%), and “high BMI trajectory” (195 females and 305 males, 19.21%). All children provided at least four BMIz values and a maximum of nine to capture the trajectories.

### Covariate Selection

A directed acyclic graph (DAG) was used to select maternal and offspring sociodemographic characteristics as covariates in the present study (**Supplemental Material, Figure S2**). Through questionnaire surveys during pregnancy, information on maternal age (continuous, year), education level (lower than middle school, high school, junior college, university, and above), parity (0, ≥1), household income (≤2,499 RMB, 2,500~4,000 RMB, >4,000 RMB), smoking (no, yes), and drinking during pregnancy (no, yes) was obtained. Information on delivery mode (cesarean section, vaginal delivery) as well as sex (male, female), birth weight (continuous, g), length of neonates (continuous, cm), and gestational age (continuous, week) was extracted from medical records. During childhood visits, information on breastfeeding pattern (exclusively breastfeeding, mixed feeding, artificial feeding) and duration (≤6 months, >6 months), daily physical activity (<1 hour, 1~2 hours, >2 hours), and dietary information (component 1~component 6) was collected.

A homemade food frequency questionnaire (26 items) was used when the children were 30 months of age. The Cronbach’s α of this scale was 0.861 (31). In brief, a six-level scoring method was used to record the children’s homemade food consumption frequency one month prior to the visit. The results of the Kaiser–Meyer–Olkin test (KMO, KMO measure of sampling adequacy = 0.881) and Bartlett’s test (Bartlett’s test of sphericity approx. chi-square = 20,013.314, *p* < 0.005) indicated that this questionnaire was suitable for factor analysis. Using the quartimax of orthogonal rotation, a total of six factors (component 1~component 6) were extracted according to the criterion that the eigenvalue was >1, and the cumulative contribution rate was 54.449%. An item with an absolute value of factor loading ≥0.3 for a principal component was considered to be well represented on that principal component (**Supplemental Material, Table S3**).

### Main Statistical Analyses

The mean ± standard deviation and numbers of cases (percentage) were used to describe continuous and categorical variables, respectively. The chi-square test for proportions and ANOVA F-test for means of demographic characteristics were used to compare among the latent BMI trajectory groups. Maternal age, education, parity, smoking and drinking during pregnancy, and household income were identified as the minimal set for necessary adjustment according to the DAG and were adjusted in the following primary analyses. The missing data on the covariates with less than 10% missing information were imputed using multiple imputation. Five sets were created. Effect estimates were pooled to obtain the overall results according to Rubin’s rules (32).

First, generalized linear regression models and binary logistic regression models were used to analyze the associations of GMS components and PRSs with BMIz scores and risks of overweight/obesity at each child visit, respectively. Tests of heterogeneity were performed by assessing the *p*-value of the interaction term between GMS components, PRSs, and child age in the regression models. Taking the correlation between multiple measurements of an individual at different ages into consideration, a generalized estimating equation (GEE) was used to analyze the marginal effects of GMS components, SNPs, and PRSs, and their interactions on the repeated measurements of the outcome variables. Link functions selected general linear regression for the continuous variable BMIz and binary logistic regression for the categorical variable overweight/obesity. In addition, gender-stratified models to evaluate the differences in predicted BMI trajectories associated with GMS, PRSs, and their interaction variables were conducted using the *loess* function with a 0.7 control smoothness parameter in Rstudio. Furthermore, multinomial logistic regression was performed to evaluate the relations of the BMIz growth trajectory groups with GMS components and PRS. Multiplicative interaction was assessed by adding an interaction variable (GMS × PRS) to the logistic regression models. Based on the results of multinomial logistic regression models, the method outlined by Andersson et al. (33) was used to test the additive interactions between GMS and PRS on the BMIz growth trajectory groups. A 2×2 factorial design was used to compute the attributable proportion due to interaction (AP), the relative excess risk due to interaction (RERI), and the synergy index (SI). If the *p*-value for AP, REMI, or SI was less than 0.05, the additive association was considered.

To test the robustness of our results, we performed some sensitivity analyses of the interaction between GMS and PRS as follows: (1) children’s breastfeeding, physical activity, and dietary information were additionally adjusted for (excluding children without this information), although these factors were measured after the GMS exposure variable and cannot be real confounders; 2) children with preterm birth and low and high birth weight were excluded because birth weight and gestational age were important factors in offspring growth; 3) a weighted PRS was calculated based on each SNP’s relative effect size (β coefficient) obtained from a GWAS (24). Similarly, all children were divided into two groups using the 75th percentile as the cutoff value.

Statistical analyses were conducted for girls and boys with SPSS software (Version 16.0), Mplus 7.0 software, Rstudio, and an Excel spreadsheet set up by Tomas Anderson, and a two-tailed *p* < 0.05 was defined as statistically significant.

## Results

### Demographic Characteristics

Maternal and children’s characteristics included and excluded in the present study are shown in **Table 1**. Maternal average age and BMI were 26.70 years and 20.84 kg/m^2^, respectively. A total of 11.95% of women were overweight or obese before pregnancy. The mean GWG was 17.81 kg, and 62.05% of women had excessive GWG. A total of 5.49% and 12.45% of women were afflicted by HDP and GDM, respectively. A total of 379 women (14.56%) and 104 women (4.00%) were classified into medium-risk and high-risk GMS groups, respectively. Of the 2,603 newborns, 1,327 (50.98%) were boys, and 1,276 (49.02%) were girls. Significant differences in maternal age, education, neonates’ gestational age, breastfeeding periods, physical activity, and dietary patterns between individuals included and excluded in the present study were shown. During childhood, 12 visits were successively performed from birth to 72 months of age. The BMI and BMIz scores at each visit are shown in the **Supplemental Material, Table S4**.

**Table 1:** Demographic characteristics of the population included and excluded in the present study.

Maternal Characteristics	Means ± SD/N(%)	
	Population Included (N = 2,603)	Population Excluded (N = 670)
**Maternal age (year)** ^a^	26.70 ± 3.60	26.23 ± 3.76
**Prepregnancy BMI (kg/m^2^)**	20.84 ± 2.82	20.96 ± 2.78
Underweight	499 (19.17%)	118 (17.61%)
Normal weight	1,793 (68.88%)	471 (70.30%)
Overweight/obesity	311 (11.95%)	81 (12.09%)
**Education** ^a^		
Lower than middle school	490 (18.82%)	170 (25.37%)
High school	585 (22.47%)	150 (22.39%)
Junior college	810 (31.12%)	206 (30.75%)
University and above	718 (27.58%)	144 (21.49%)
**Parity**		
0	2,319 (89.09%)	579 (86.42%)
≥1	284 (10.91%)	91 (13.58%)
**Household income monthly (RMB)**		
≤2,499	699 (26.85%)	168 (25.07%)
2,500~4,000	1,101 (42.30%)	301 (44.93%)
>4,000	803 (30.85%)	201 (30.00%)
**Smoking during pregnancy**		
No	2,598 (99.81%)	669 (99.85%)
Yes	5 (0.19%)	1 (0.15%)
**Drinking during pregnancy**		
No	2,392 (91.89%)	620 (92.54%)
Yes	211 (8.11%)	50 (7.46%)
**Gestational weight gain (kg)**	17.82 ± 5.05	17.84 ± 5.24
Insufficient	227 (8.84%)	54 (8.31%)
Adequate	748 (29.12%)	184 (28.31%)
Excessive	1,594 (62.05%)	412 (63.38%)
**Diabetes mellitus (DM)** ^a^		
Never	2,279 (87.55%)	562 (83.88%)
Gestational DM	324 (12.45%)	95 (14.18%)
DM complicated with pregnancy	0 (0.00%)	13 (1.94%)
**Hypertensive disorders of pregnancy** ^a^		
Never	2460 (94.51%)	606 (91.40%)
Hypertensive disorder of pregnancy	143 (5.49%)	51 (1.66%)
Chronic hypertension	0 (0.00%)	6 (0.90%
**Cesarean section**		
Yes	1,296 (49.79%)	326 (48.66%)
No	1,307 (50.21%)	344 (51.34%)
**Gestational metabolic syndrome**		
No risk	799 (30.70%)	—
Low risk	1,321 (50.75%)	—
Medium risk	379 (14.56%)	—
High risk	104 (4.00%)	—
**Child characteristics**	**Means ± SD/N (%)**	
**Gender**		
Male	1,327 (50.98%)	339 (51.05%)
Female	1,276 (49.02%)	325 (48.95%)
**Birth weight (g)**	3,368.89 ± 436.97	3351.77 ± 486.45
**Gestational age (week)** ^a^	39.48 ± 1.30	39.30 ± 1.53
**Breastfeeding pattern**		
Exclusive breastfeeding	235 (11.83%)	50 (8.96%)
Mixed feeding	1,149 (57.85%)	337 (60.39%)
Artificial feeding	602 (30.31%)	171 (30.65%)
**Breastfeeding periods^a^**		
≤6 month	579 (28.98%)	183 (35.74%)
>6 month	1,419 (71.02%)	329 (64.26%)
**Daily physical activity^a^**		
<1 hour	481 (24.77%)	73 (17.38%)
1~2 hours	703 (36.20%)	223 (53.10%)
>2 hours	758 (39.03%)	124 (29.52%)
**Dietary information**		
Component 1 ^a^	−0.013 ± 0.993	0.081 ± 1.017
Component 2	−0.019 ± 0.988	0.024 ± 1.083
Component 3	−0.016 ± 0.969	0.073 ± 1.158
Component 4	0.011 ± 0.982	−0.069 ± 1.066
Component 5	0.002 ± 1.005	0.051 ± 1.043
Component 6 ^a^	0.030 ± 0.989	−0.100 ± 1.029

**^a^** Difference was found between populations included and excluded in the present study, p-value < 0.05.

### Association Between GMS Components and PRS and Measurements of BMIz and Overweight/Obesity at Each Visit During Childhood

Significant relationships between GMS components and PRS and BMIz scores and risks of overweight/obesity were revealed in both girls and boys, especially during later childhood (**Supplemental material, Figures S3–S6**). At most visits, the BMIz scores and risks of overweight/obesity in children born to underweight mothers decreased, whereas those born to overweight/obese mothers increased, compared with girls and boys born to mothers with normal weight. The greater maternal GMS risk scores were, the higher children’s BMIz scores and risks of overweight/obesity were. In girls, high PRSs were also positively associated with their BMIz scores and risks of overweight/obesity. In boys, maternal GDM status was associated with increased BMIz scores and risks of overweight/obesity (**Supplemental Material, Figures S3–S6**). Some heterogeneity among these relationships in terms of child age was observed (**Supplemental Material, Figures S3–S6**).

### Association Between GMS Components and Repeated Measurements of BMIz and Overweight/Obesity

Compared with children born to mothers with normal weight, the BMIz scores of children born to underweight mothers decreased, both in girls and boys, whereas those born to overweight/obese mothers increased their BMIz scores (**Table 2**). Similarly, the BMIz of girls and boys born to mothers with excessive GWG increased compared with children born to mothers with adequate GWG (**Table 2**). GDM was positively associated with BMIz in both girls and boys. The BMIz of children increased with the increasing GMS risk scores (**Table 2**). Consistent with these results, positive correlations were also revealed between prepregnancy overweight/obesity, excessive GWG, GDM, HDP, low to high risks of GMS, and children’s prevalence of overweight/obesity (**Table 2**).

**Table 2:** Association of gestational metabolic syndrome parameters with children’s BMIz scores and overweight/obesity using a generalized estimating equation.

Variables	Association of BMIz Score [β (95%CI)]		Association of Overweight/Obesity [OR (95%CI)]	
	In girls	In boys	In girls	In boys
Prepregnancy BMI				
Underweight	**−0.261 (−0.351, −0.170)**	**−0.435 (−0.562, −0.308)**	**0.559 (0.424, 0.739)**	**0.518 (0.408, 0.658)**
Normal weight	Reference	Reference	Reference	Reference
Overweight/obesity	**0.286 (0.177,0.395)**	**0.327 (0.143, 0.511)**	**1.740 (1.338, 2.263)**	**1.697 (1.327, 2.170)**
Gestational weight gain				
Insufficient	**−**0.073 (**−**0.239, 0.093)	**−**0.200 (**−**0.450, 0.051)	1.090 (0.713, 1.668)	0.717 (0.502, 1.023)
Adequate	Reference	Reference	Reference	Reference
Excessive	**0.223 (0.125, 0.320)**	**0.173 (0.028, 0.318)**	**1.568 (1.264, 1.945)**	**1.474 (1.212, 1.793)**
GDM				
Yes	**0.123 (0.009, 0.237)**	**0.279 (0.072, 0.486)**	**1.357 (1.043, 1.764)**	**1.803 (1.425, 2.282)**
No	Reference	Reference	Reference	Reference
HDP				
Yes	**−**0.057 (**−**0.266,0.152)	**−**0.057 (**−**0.327,0.212)	1.115 (0.741,1.678)	**1.487 (1.064,2.078)**
No	Reference	Reference	Reference	Reference
GMS				
No risk	Reference	Reference	Reference	Reference
Low risk	**0.167 (0.089, 0.246)**	**0.180 (0.039, 0.321)**	**1.424 (1.139, 1.779)**	**1.519 (1.241, 1.859)**
Medium risk	**0.351 (0.235, 0.466)**	**0.381 (0.145, 0.617)**	**2.300 (1.751, 3.021)**	**2.052 (1.554, 2.708)**
High risk	**0.431 (0.244, 0.618)**	**0.582 (0.248, 0.917)**	**2.024 (1.241, 3.300)**	**3.959 (2.769, 5.661)**
PRS	**−**0.005 (**−**0.080,0.070)	0.038 (**−**0.017,0.094)	1.058 (0.998,1.122)	0.979 (0.926, 1.034)
Low risk	Reference	Reference	Reference	Reference
High risk	0.165 (**−**0.147, 0.478)	0.114 (**−**0.110, 0.337)	**1.368 (1.072, 1.746)**	1.003 (0.785, 1.281)

BMI, body mass index; GDM, gestational diabetes mellitus; HDP, hypertensive disorders of pregnancy; GMS, gestational metabolic syndrome; PRS, polygenic risk scores.

Adjustment for maternal age, education, parity, smoking and drinking during pregnancy, and household income; bold type represents a p-value less than 0.05.

### Relationship of SNPs and PRS With Repeated Measurements of BMIz and Overweight/Obesity

Genotypes of rs12041852, rs13130484, and rs3829849 were significantly associated with BMIz among girls (**Supplemental Material, Table S5**). Compared with children homozygous for the wild type, children carrying mutant homozygosity of rs13130484 and rs1421085 had a higher risk of overweight/obesity (**Supplemental Material, Table S5**). The rs12041852 genotype was associated with BMIz score and an increased risk of overweight/obesity in boys (**Supplemental Material, Table S5**). Compared with children with low PRS risk, girls with high PRS risks had an increased risk of overweight/obesity (**Table 2**).

### Interaction Between GMS and PRS on Repeated Measurements of BMIz and Overweight/Obesity

Due to the limited sample size, to analyze the effect of the interaction between GMS and PRS on BMIz and overweight/obesity prevalence, GMS was divided into a risk-free (no GMS risk) group and a risky group (low to high GMS risk). **Table 3** shows the results of the analysis in which we tested for additive and multiplicative interactions between GMS and PRS. Compared with children with low PRS risk and born to risk-free GMS mothers, there was an increase of 0.462 (95% CI = 0.268–0.656) in BMIz and a 1.482-fold increased risk of overweight/obesity (95% CI = 1.710–3.603) for high PRS risk in girls who were born to women with GMS risk. There was a multiplicative (both *p* < 0.001) but not an additive interaction between GMS and PRSs. Compared to the reference group of boys with low PRSs born to women free of risk of GMS, an increase of a 0.575-fold increased risk of overweight/obesity (95% CI = 1.132–2.190) for high PRSs in boys who were born to women at risk of GMS was observed. A multiplicative interaction (*p* = 0.004) was detected. The sensitivity analyses found similar relationships between the effect of GMS and PRS on repeated measurements of BMIz and overweight/obesity by additionally adjusting for breastfeeding, physical activity, and dietary pattern or by excluding children with preterm birth and low and high birth weight (**Supplemental Material, Table S7**).

**Table 3:** Association of the interaction of GMS risk and PRS risk with overweight/obesity using a generalized estimating equation.

GMS Risk	PRS Risk	Association of BMIz Score		Association of Overweight/Obesity	
In the population of Girls		β (95%CI)	β (95%CI)^a^	OR (95%CI)	OR (95%CI)^a^
Risk-free	Low risk	0 (Reference)	0 (Reference)	1.00 (Reference)	1.00 (Reference)
Risk-free	High risk	**0.218 (0.034, 0.403)**	**0.282 (0.089, 0.474)**	**1.876 (1.243, 2.830)**	**1.904 (1.269, 2.858)**
Risky	Low risk	**0.235 (0.104, 0.367)**	**0.245 (0.095, 0.395)**	**1.887 (1.350, 2.638)**	**1.887 (1.345, 2.645)**
Risky	High risk	**0.407 (0.246, 0.568)**	**0.462 (0.268, 0.656)**	**2.411 (1.659, 3.504)**	**2.482 (1.710, 3.603)**
Additive interaction:					
AP (95%CI)		−0.004 (−0.204, 0.196)	−0.010 (−0.233, 0.212)	−0.146 (−0.543, 0.251)	−0.125 (−0.510, 0.260)
RERI (95%CI)		−0.006 (−0.306, 0.294)	−0.016 (−0.369, 0.336)	−0.352 (−1.291, 0.588)	−0.309 (−1.249, 0.630)
Synergy index (95%CI)		0.988 (0.547, 1.785)	0.973 (0.540, 1.753)	0.800 (0.459, 1.396)	0.827 (0.480, 1.425)
Multiplication interaction:					
P for interaction		**<0.001**	**<0.001**	**<0.001**	**<0.001**
**In the population of Boys**		**β (95%CI)**	**β (95%CI)** ^a^	**OR (95%CI)**	OR (95%CI)^a^
Risk-free	Low risk	0 (Reference)	0 (Reference)	1.00 (Reference)	1.00 (Reference)
Risk-free	High risk	−0.052 (−0.343, 0.240)	−0.181 (−0.555, 0.193)	1.018 (0.646, 1.604)	0.994 (0.638, 1.550)
Risky	Low risk	0.029 (−0.176, 0.234)	−0.099 (−0.354, 0.156)	**1.570 (1.184, 2.082)**	**1.543 (1.166, 2.044)**
Risky	High risk	0.175 (−0.083, 0.433)	0 (−0.309, 0.309)	**1.618 (1.163, 2.250)**	**1.575 (1.132, 2.190)**
Additive interaction:					
AP (95%CI)		0.178 (−0.126, 0.483)	0.260 (−0.143, 0.663)	0.018 (−0.373, 0.410)	0.024 (−0.361, 0.408)
RERI (95%CI)		0.212 (−0.160, 0.585)	0.260 (−0.139, 0.659)	0.030 (−0.605, 0.665)	0.037 (−0.569, 0.644)
Synergy index (95%CI)		−9.001 (—,—)	0 (—,—)	1.050 (0.359, 3.076)	1.069 (0.348, 3.284)
Multiplication interaction:					
P for interaction		0.457	0.705	**0.003**	**0.004**

a Adjustment for maternal age, education, parity, smoking and drinking during pregnancy, and household income; bold type represents a p-value less than 0.05.

### GMS, PRS, and Their Interaction Effects on BMI Trajectory

**Figure 1:**
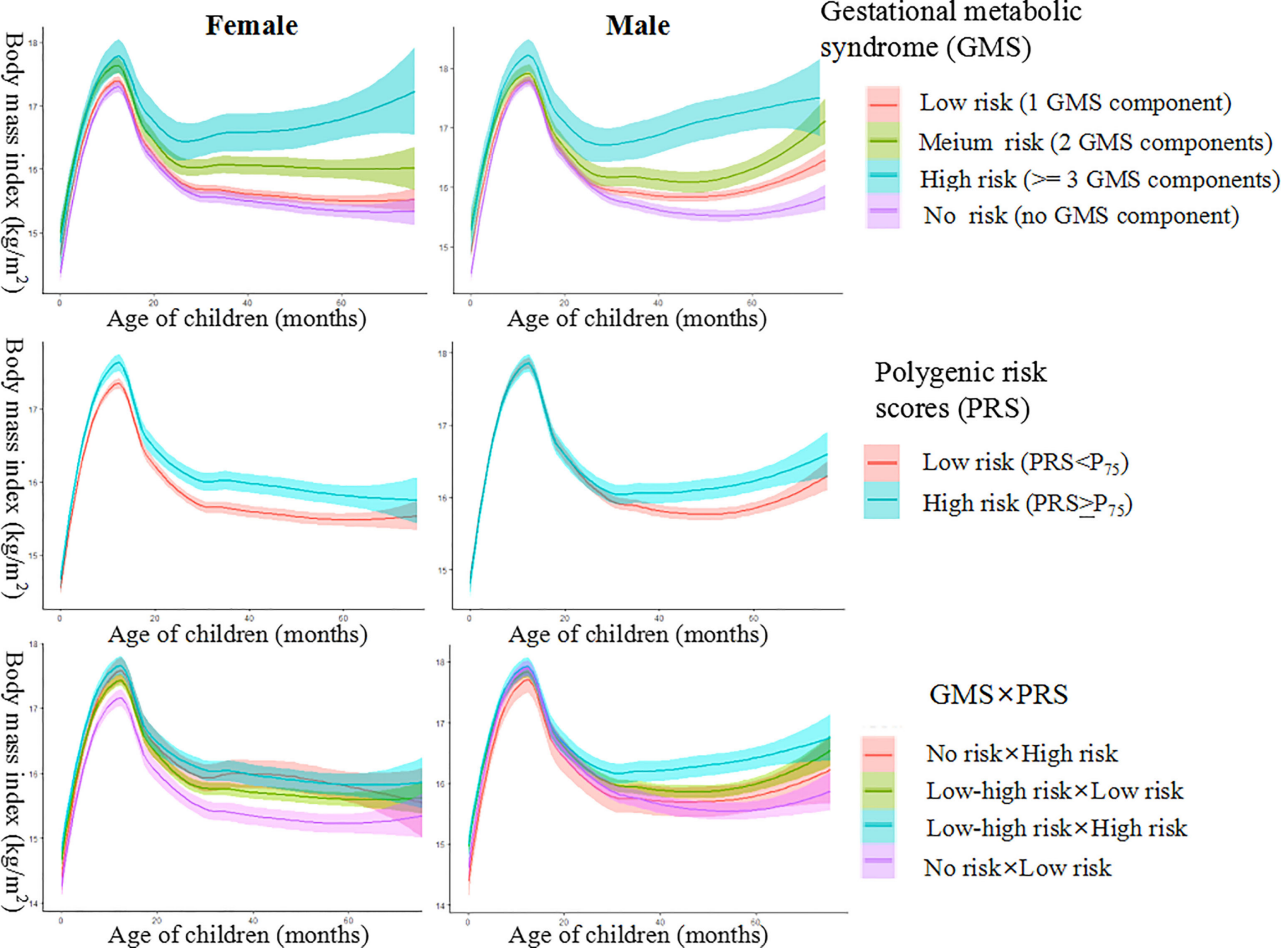
Female and male body mass index (BMI) trajectory for groups of gestational metabolic syndrome risk, polygenic risk, and their interaction.

In both girls and boys, individuals in the GMS high-risk group were prone to be stable at the highest trajectory, and those in the GMS risk-free group were prone to be at the lowest trajectory (**Figure 1**). The BMI trajectory of children in the PRS high-risk group was higher than those in the PRS low-risk group. The BMI trajectory of girls in the GMS risk-free × PRS low-risk group remained constantly at the lowest trajectory, whereas the BMI trajectory of boys in the GMS risky × PRS high-risk group remained the highest (**Figure 1**).

### Relation Between GMS Components and BMIz Latent Trajectory

Compared with girls with a low BMIz trajectory, girls born to mothers with prepregnancy overweight/obesity or excessive GWG showed higher risks of being in the high BMIz trajectory (**Table 4**). Girls with medium and high risks of GMS also had a higher probability of being in the high BMIz trajectory (**Table 4**). In boys, children born to prepregnancy underweight mothers or mothers with insufficient GWG had a lower risk of being on medium and high BMIz trajectories than low BMIz trajectories (**Table 4**).

**Table 4:** Association of gestational metabolic syndrome parameters with children’s BMIz score trajectory group.

Variables	Girls		Boys	
	Medium BMIz Trajectory	High BMIz Trajectory	Medium BMIz trajectory	High BMIz Trajectory
	*OR(95%CI)^a^*	*OR(95%CI)^a^*	*OR(95%CI)^a^*	*OR(95%CI)^a^*
Prepregnancy BMI				
Underweight	**0.722 (0.526, 0.991)**	**0.609 (0.375, 0.987)**	**0.650 (0.469, 0.900)**	**0.439 (0.287, 0.672)**
Normal weight	Reference	Reference	Reference	Reference
Overweight or obesity	1.432 (0.935, 2.193)	**2.224 (1.319, 3.752)**	1.145 (0.722, 1.816)	1.222 (0.726, 2.057)
Gestational weight gain				
Insufficient	0.884 (0.545, 1.436)	0.947 (0.458, 1.960)	**0.607 (0.382, 0.965)**	**0.490 (0.271, 0.888)**
Adequate	Reference	Reference	Reference	Reference
Excessive	1.107 (0.837, 1.463)	**1.533 (1.023, 2.297)**	1.021 (0.751, 1.388)	1.051 (0.733, 1.508)
Gestational diabetes mellitus				
Yes	1.149 (0.779, 1.695)	1.307 (0.777, 2.198)	0.847 (0.558, 1.286)	1.143 (0.713, 1.832)
No	Reference	Reference	Reference	Reference
Hypertensive disorders of pregnancy				
Yes	0.959 (0.548, 1.678)	0.643 (0.324, 1.279)	0.794 (0.424, 1.488)	0.902 (0.427, 1.906)
No	Reference	Reference	Reference	Reference
Gestational metabolic syndrome				
No risk	Reference	Reference	Reference	Reference
Low risk	1.103 (0.830, 1.001)	1.285 (0.843, 1.957)	1.006 (0.745, 1.361)	1.055 (0.735, 1.513)
Medium risk	1.357 (0.909, 2.026)	**2.488 (1.465, 4.226)**	1.160 (0.732, 1.837)	1.311 (0.767, 2.240)
High risk	1.717 (0.800, 3.686)	**2.909 (1.142, 7.414)**	1.522 (0.691, 3.356)	2.169 (0.916, 5.134)
PRS	1.022 (0.941, 1.111)	1.042 (0.927, 1.171)	0.947 (0.863, 1.040)	0.898 (0.803,1.004)
Low risk	Reference	Reference	Reference	Reference
High risk	1.290 (0.893, 1.863)	**1.756 (1.068, 2.887)**	0.919 (0.621, 1.358)	0.860 (0.540, 1.369)

a Adjustment for maternal age, education, parity, smoking and drinking during pregnancy, and household income; bold type represents p-values less than 0.05.

### Relation Between SNPs and BMIz Latent Trajectory

Significant associations between genotypes of rs13130484 and high BMIz trajectories were observed in girls, whereas genotypes of rs12041852 and rs8092503 were associated with medium or high BMIz trajectories in boys (**Supplemental Material, Table S7**). Compared with the PRS low-risk group, only girls with high PRS risk were linked to an increased risk of high BMIz trajectories (**Table 4**).

### Effect of the Interaction Between GMS and PRS on BMIz Latent Trajectory

Compared to the reference group of girls with low PRS risk born to women with no risk of GMS, a 0.761-fold increased risk of being in the medium BMIz trajectory (95% CI = 1.053–9.247) was observed for high PRS risk in girls who were born to women at risk of GMS. There was a multiplicative (*p* = 0.033) but not an additive interaction between GMS and PRS. There was a 2.337-fold increased risk of being in the high BMIz trajectories (95% CI = 2.202–5.507) for high PRSs in girls who were born to women at risk of GMS compared to the reference group of girls with low PRSs born to women with no risk of GMS. There was a multiplicative (*p* = 0.002) but not an additive interaction between GMS and PRS (**Table 5**).

**Table 5:** Association of the interaction of GMS risk and PRS risk with children’s BMIz score trajectory group.

GMS Risk	PRS Risk	Medium BMIz Trajectory		High BMIz Trajectory	
In the population of Girls		OR(95%CI)	OR(95%CI)^a^	OR(95%CI)	OR(95%CI)^a^
Risk-free	Low risk	1.00 (Reference)	1.00 (Reference)	1.00 (Reference)	1.00 (Reference)
Risk-free	High risk	1.603 (0.867, 2.962)	1.629 (0.878, 3.022)	**2.096 (1.277, 3.441)**	**2.128 (1.291, 3.507)**
Risky	Low risk	1.502 (0.994, 2.269)	**1.536 (1.012, 2.330)**	**2.083 (1.076, 4.035)**	**2.056 (1.481, 2.855)**
Risky	High risk	**1.721 (1.034, 2.862)**	**1.762 (1.053, 2.947)**	**3.243 (1.543, 6.818)**	**3.337 (2.202, 5.057)**
Additive interaction:					
AP (95%CI)		−0.222 (−0.950, 0.505)	−0.229 (−0.962, 0.503)	0.020 (−0.424, 0.464)	0.046 (−0.396, 0.487)
RERI (95%CI)		−0.383 (−1.590, 0.824)	−0.404 (−1.642, 0.835)	0.065 (−1.387, 1.518)	0.153 (−1.350, 1.655)
Synergy index (95%CI)		0.653 (0.191, 2.239)	0.654 (0.196, 2.179)	1.030 (0.533, 1.989)	1.070 (0.548, 2.088)
Multiplication interaction:					
P for interaction		**0.033**	**0.027**	**0.002**	**0.002**
**In the population of Boys**		**OR ( (95%CI)**	**OR ( (95%CI)**^a^	**OR ( (95%CI)**	**OR ( (95%CI)**^a^
Risk-free	Low risk	1.00 (Reference)	1.00 (Reference)	1.00 (Reference)	1.00 (Reference)
Risk-free	High risk	0.532 (0.268, 1.057)	0.532 (0.264, 1.072)	0.628 (0.269, 1.470)	0.598 (0.322, 1.077)
Risky	Low risk	**0.517 (0.319, 0.838)**	**0.549 (0.336, 0.898)**	0.815 (0.457, 1.452)	0.850 (0.556, 1.301)
Risky	High risk	0.613 (0.348, 1.081)	0.649 (0.364, 1.157)	0.829 (0.420, 1.636)	0.851 (0.518, 1.398)
Additive interaction:					
AP (95%CI)		**0.919 (0.068, 1.771)**	**0.876 (0.055, 1.698)**	0.465 (−0.153, 1.084)	0.473 (−0.133, 1.079)
RERI (95%CI)		**0.564 (0.126, 1.002)**	**0.569 (0.112, 1.025)**	0.386 (−0.098, 0.870)	0.403 (−0.085, 0.890)
Synergy index (95%CI)		**0.407 (0.196, 0.843)**	**0.381 (0.160, 0.909)**	0.306 (0.046, 2.030)	0.270 (0.027, 2.695)
Multiplication interaction:					
P for interaction		0.838	0.089	0.960	0.805

AP, the attributable proportion due to interaction; RERI, the relative excess risk due to interaction; Normative BMI trajectory was referred to as the reference group.

a Adjustment for maternal age, education, parity, smoking and drinking during pregnancy, and household income; bold type represents p-values less than 0.05.

Among boys, we did not observe an increase in the risk of being in the medium and high BMIz trajectories with high PRSs for those who were born to women at risk of GMS compared to boys with low PRSs born to women with no risk of GMS. However, we observed a significant additive interaction between the GMS risk and PRS high-risk groups, with the proportion of risk of being in the medium BMIz trajectories due to the additive interaction (AP) of 0.876 (95% CI = 0.0554–1.698) (**Table 5**).

Sensitivity analyses found similar effects of the interaction between GMS and PRS on BMIz latent trajectory by additionally adjusting for breastfeeding, physical activity, and dietary pattern or by excluding children with preterm birth and low and high birth weight (**Supplemental Material, Table S8**). When a weighted PRS (PRS_β_) was calculated according to each SNP relative effect size (β coefficient), similar results were obtained (**Supplemental Material, Table S9**).

## Discussion

In the present study, both boys and girls born to mothers with prepregnancy overweight/obesity, excessive GWG, or GDM (single- or coexisting) had increased BMIz scores and risks of overweight/obesity. The PRS was positively associated with the risk of overweight/obesity among girls. Maternal GMS significantly interacted with the child’s PRS on BMIz scores and the risk of overweight/obesity among girls. Hierarchical BMI trajectory graphs showed consistent findings in both boys and girls, who were divided into three groups. Only among girls were both the main effects of GMS and PRS and their interactions observed in the associations with preschoolers’ BMI trajectories; among girls, the higher the GMS risk or PRS they had, the higher probability of being in a high BMIz trajectory. The similar results of several sensitivity analyses showed the robustness of our findings.

A number of previous studies have investigated the relationship between maternal prepregnancy obesity and pregnancy complications and obesity in childhood. However, most of them were focused on the absolute level of BMI or risk of overweight/obesity at a specific age (34, 35). While children with different BMI trajectories may have different risks for subsequent health problems, it is important to analyze the potential heterogeneity of BMI changes over a long period (e.g., childhood). Recent progress in statistical methodology has made this possible. Additionally, very few surveys have assessed the cumulative effects of maternal metabolic risk factors but a single exposure effect to one of them (e.g., GDM, GWG, and HDP in a single statistical model) on childhood BMI trajectory (36, 37). A cohort study including 734 mothers and their children from birth to 4~5 years of age suggested that the effects of maternal GDM, prepregnancy obesity, and GWG on children’s BMI trajectories differed in effect size, timing, and direction (36). Furthermore, a significant effect of the interaction between GDM and preeclampsia on the BMI trajectory of children was revealed (38). Therefore, studies on the cumulative effects of multiple maternal MS components on child health are needed to precisely identify high-risk individuals. Hu et al. (2019) identified three discrete BMI trajectory groups among 1,425 children from 0~4 years of age (five physical examinations) and found that the risk of being in an increasingly high BMI trajectory was higher among children born to mothers with more than one metabolic risk factor, including prepregnancy obesity, GWG, and GDM (15). These results were consistent with our findings, yet we doubled the sample size (N = 2,603), increased the frequency (12 physical examinations) and length of follow-up in childhood (0–6 years of age), and expanded the range of maternal metabolic risk factors by adding HDP. To our surprise, although a previous study indicated that obesity along with HDP and GDM is the central attribute of the MS that may occur in pregnancy (14), our results showed that prepregnancy BMI and GWG outweigh the impacts of GDM and HDP on children’s long-term BMI trajectories, suggesting that the effect of GDM and HDP on children’s high BMI trajectory and obesity status might be partially influenced by maternal prepregnancy obesity and excessive GWG (39). Thus, our study emphasized the significance of weight management before and during pregnancy regarding obesity prevention for the next generation.

Furthermore, our present study revealed a sex-specific relationship between GMS status and BMI trajectory, and maternal GMS seemed to exert a more long-lasting effect on the girls’ BMI. Pregnancy is a critical window when an altered intrauterine environment characterized by GDM or maternal obesity can cause permanent changes in cellular function (40). Cumulative evidence indicates that different responses of males and females to adverse intrauterine environments such as maternal obesity could lead to different risks of metabolic disease later in their life (41, 42). And the core mechanisms underlying this may be sex differences in placental development such as epigenetic placental gene regulation and function (43–45). Effective management of prenatal obesity and maternal metabolic risks during pregnancy may prevent or slow the development of childhood obesity. If the windows of prepregnancy and pregnancy to provide related interventions are missed, girls who are born to mothers at risk of GMS will be more likely to be obese, which is supported by the evidence of the present study.

As far as we know, this is the first study to indicate that the association of maternal GMS risk with obesity and BMI trajectories in early childhood may differ according to children’s PRS status. Previously, only one study found that maternal genetic predisposition of BMI interacted with GDM status on offspring childhood overweight and obesity status but could not exclude the possibility that children’s polygenic susceptibility was attributed to the observed relationships (46). Interestingly, our study indicated that children’s genetic susceptibility did play a role. The underlying mechanisms were unclear. To the best of our knowledge, maternal nutritional disturbance is one of the most important fetal programming stimuli. For instance, fetal exposure to diabetes and diabetes-related metabolic derangements may alter the epigenome of the fetus and the reproductive cells and modulate the epigenome transgenerationally, thus potentially increasing offspring susceptibility to chronic diseases (47), which fits the Developmental Origins of Health and Disease (DOHaD) hypothesis. To date, there is strong evidence that an adverse intrauterine and perinatal environment resulting in epigenetic modifications could reveal the possible pathophysiological mechanisms for long-term disease development in offspring (48). Although genetic effects are difficult to change, this study found that GMS and PRS exhibited an interactive effect on offspring obesity, and their population attributable rate was only slightly lower than that of independent GMS exposure. Therefore, our study suggested that children exposed to maternal GMS were at higher risk of obesity, especially those with genetic susceptibilities, and hierarchical management should be delivered.

There are some potential limitations that should be considered. First, although some important confounders, including breastfeeding, diet, and physical activity, were adjusted for, paternal risk factors could not be assessed because of missing information. Second, maternal self-reported prepregnancy body weight was used to calculate maternal BMI, which might have introduced bias. However, a strong correlation between maternal self-reported prepregnancy body weight and the measured body weight at enrollment at an average of 10 gestational weeks was observed (Pearson’s *r* = 0.914). These results indicated that this was not a major source of bias in this study. Third, we applied GMS status as one integrated index in statistical analyses to avoid multiple comparisons to improve statistical power, but data on blood lipids, one of the core components of GMS, were unavailable in the present study. Additionally, a total of 670 children (20.47%) were lost to follow-up, which might have resulted in some bias in the statistical results. Finally, 43.05% of mother-child pairs were excluded from the statistical models of PRS and its interaction with GMS due to missing cord blood samples. We evaluated and found little difference in the demographic characteristics between populations with and without gene data.

In summary, maternal GMS status increased the BMIz scores and the risk of obesity in both boys and girls and elevated the child’s BMI trajectory from birth to six years old among girls. Only among girls was PRS significantly associated with BMI trajectory and the risk of obesity and modified the associations between maternal GMS status and obesity biomarkers. Thus, regarding childhood obesity prevention, steps should be taken to decrease maternal metabolic risks before and during pregnancy, and sex discrepancies should be noted to identify high-risk populations after birth to hierarchically manage them.

## Data Availability Statement

The original contributions presented in the study are included in the article/**Supplementary Material**. Further inquiries can be directed to the corresponding author.

## Ethics Statement

The studies involving human participants were reviewed and approved by The Ethics Committee of Anhui Medical University. The patients/participants provided their written informed consent to participate in this study.

## Author Contributions

Writing-original draft and formal analysis: B-bZ and HG; Investigation and data collection: M-lG, JT, X-lW, L-hW, S-yZ, FD, and HG; Methodology and project administration: KH and X-yW; Supervision and writing-review and editing: PZ and F-bT; Conceptualization and validation: F-bT; Funding acquisition: B-bZ, HG, and F-bT. All authors have agreed to authorship and order of authorship for this manuscript. All authors contributed to the article and approved the submitted version.

## Funding

The study is funded by the National Key Research and Development Project (No. 2018YFC1004200), the National Natural Science Foundation of China (No. 82073564 and No. 82103856), and the Provincial Natural Science Foundation of Anhui (No. 2108085QH359).

## Conflict of Interest

The authors declare that the research was conducted in the absence of any commercial or financial relationships that could be construed as a potential conflict of interest.

## Publisher’s Note

All claims expressed in this article are solely those of the authors and do not necessarily represent those of their affiliated organizations, or those of the publisher, the editors and the reviewers. Any product that may be evaluated in this article, or claim that may be made by its manufacturer, is not guaranteed or endorsed by the publisher.
