# Supplementary material for: Sex Discrepancy Observed for Gestational Metabolic Syndrome Parameters and Polygenic Risk Associated With Preschoolers’ BMI Growth Trajectory: The Ma’anshan Birth Cohort Study
Source: Front Endocrinol (Lausanne). 2022 Jul 1;13:857711. doi: 10.3389/fendo.2022.857711 (PMC9283700; doi:10.3389/fendo.2022.857711)
Supplement: Supplementary file 1 [file DataSheet_1.docx]

**Supplemental Material**

**Sex discrepancy observed for gestational metabolic syndrome parameters and polygenic risk associated with preschoolers’ BMI growth trajectory: the Ma’anshan Birth Cohort study**

Bei-bei Zhu ^1,2✝^; Hui Gao ^3,4✝^; Meng-long Geng ^1,5^; Xiu-long Wu ^1,2^; Juan Tong ^1,5^; Fen Deng ^1,5^; Si-ying Zhang ^1^; Li-hong Wu ^1^; Kun Huang ^1,2,5^; Xiao-yan Wu ^1,2,5^; Hong Gan ^1,2^; Peng Zhu ^1,2,5^; Fang-biao Tao ^1,2,5,*^

^1^ Department of Maternal, Child and Adolescent Health, School of Public Health, Anhui Medical University, No 81 Meishan Road, Hefei 230032, Anhui, China

^2^ Key Laboratory of Population Health Across Life Cycle (Anhui Medical University), Ministry of Education of the People’s Republic of China, No. 81 *Meishan* Road, Hefei 230032, Anhui, China

^3^ Department of Pediatrics, the First Affiliated Hospital of Anhui Medical University, No.218 Jixi Road, Hefei 230022, Anhui, China

^4^ NHC Key Laboratory of Study on Abnormal Gametes and Reproductive Tract, No 81 Meishan Road, Hefei 230032, Anhui, China

^5^Anhui Provincial Key Laboratory of Population Health and Aristogenics, Anhui Medical University, No 81 Meishan Road, Hefei 230032, Anhui, China

^✝^ BB Zhu and H Gao worked equally to this work.

**Correspondence:**

taofangbiao@126.com.

**Table S1.** Recommendations for the total weight gain and the rate of weight gain during pregnancy ^a^

| **Prepregnancy BMI categories by WGOC** | **Prepregnancy BMI categories by WHO** | **Recommendations for the total weight gain by IOM (kg)** | **Recommendations for the rate of weight gain during the second and third trimesters of pregnancy (kg/week)** |
| --- | --- | --- | --- |
| Underweight | <18.5 kg/m^2^ | 12.5-18.0 | 0.45 (0.45-0.60) |
| Normal weight | 18.5-24.9 kg/m^2^ | 11.5-16.0 | 0.45(0.36-0.45) |
| Overweight | 25.0-29.9 kg/m^2^ | 7.0-11.5 | 0.27(0.23-0.32) |
| Obese | >30.0 kg/m^2^ | 5.0-9.0 | 0.23(0.18-0.27) |

Abbreviations: BMI, body mass index; WGOC, Working Group on Obesity in China; WHO, World Health Organization; IOM, Institute of Medicine.

^a^ Rasmussen KM, Yaktine AL. Weight Gain During Pregnancy: Reexamining the Guidelines. Washington (DC): National Academies Press (US), (2009)

**Table S2.** Demographic characteristics of the population with and without gene data

|  | **Mean±SD/N(%)** | |
| --- | --- | --- |
| **Maternal characteristics** | **Population with gene data (N = 1 482)** | **Population without gene data (N = 1 121)** |
| **Maternal age (year)** | 26.74±3.55 | 26.66±3.68 |
| **Prepregnancy BMI (kg/m^2^)** | 20.81±2.76 | 20.88±2.89 |
| Underweight | 283(19.10%) | 216(19.27%) |
| Normal weight | 1024(69.10%) | 769(68.60%) |
| overweight/obese | 175(11.81%) | 136(12.13%) |
| **Education** |  |  |
| Lower than middle school | 257(17.34%) | 233(20.79%) |
| High school | 343(23.14%) | 242(21.59%) |
| Junior college | 459(30.97%) | 351(31.31%) |
| University and above | 423(28.54%) | 295(26.32%) |
| **Parity** |  |  |
| 0 | 1321(89.14%) | 998(89.03%) |
| ≥1 | 161(10.86%) | 123(10.97%) |
| **Household income monthly (RMB)** | |  |
| ≤2499 | 390(26.32%) | 309(27.56%) |
| 2500～4000 | 618(41.70%) | 483(43.09%) |
| ＞4000 | 474(31.98%) | 329(29.35%) |
| **Smoking during pregnancy** |  |  |
| No | 1480(99.87%) | 1118(99.73%) |
| Yes | 2(0.13%) | 3(0.27%) |
| **Drinking during pregnancy** |  |  |
| No | 1375(92.78%) | 1017(90.72%) |
| Yes | 106(7.15%) | 101(9.01%) |
| **Gestational weight gain (kg)** | 17.74±4.94 | 17.91±5.21 |
| Insufficient | 132(8.91%) | 99(8.85%) |
| Adequate | 446(30.09%) | 314(28.06%) |
| Excessive | 904(61.00%) | 706(63.09%) |
| **Hypertensive disorders of pregnancy** | |  |
| Yes | 71(4.79%) | 73(6.42%) |
| No | 1411(95.21%) | 1049(93.58%) |
| **Gestational diabetes mellitus** |  |  |
| Yes | 170(11.47%) | 154(13.74%) |
| No | 1312(88.53%) | 967(86.26%) |
| **Cesarean section^a^** |  |  |
| Yes | 712(48.04%) | 584(52.10%) |
| No | 770(51.96%) | 537(47.90%) |
| **Gestational metabolic syndrome ^a^** | |  |
| No risk | 479(32.32%) | 320(28.55% |
| Low risk | 751(50.67%) | 570(50.85%) |
| Medium risk | 194(13.09%) | 185(16.50%) |
| High risk | 58(3.91%) | 46(4.10%) |
| **Child characteristics** | **Mean±SD/N(%)** |  |
| **Gender** |  |  |
| Male | 748(50.47%) | 579(50.65%) |
| Female | 734(49.53%) | 542(48.35%) |
| **Birth weight (g)** | 3370.87±426.05 | 3366.27±451.20 |
| **Gestational age (week) ^a^** | 39.58±1.22 | 39.36±1.38 |
| **Breastfeeding pattern ^a^** |  |  |
| Exclusive breastfeeding | 132(12.39%) | 103(11.18%) |
| Mixed feeding | 620(58.22%) | 529(57.44%) |
| Artificial feeding | 313(29.39%) | 289(31.38%) |
| **Breastfeeding periods ^a^** |  |  |
| ≤6 month | 300(28.07% | 279(30.03%) |
| ＞6 month | 769(71.94%) | 650(69.97%) |
| **Daily body activity** |  |  |
| ＜1 hour | 238(22.95%) | 243(26.85%) |
| 1～2 hours | 390(37.61%) | 313(34.59%) |
| ＞2 hours | 409(39.44%) | 349(38.56%) |
| **Dietary pattern** |  |  |
| Component 1 | -0.010±0.979 | -0.016±1.011 |
| Component 2 | -0.042±0.961 | 0.011±1.022 |
| Component 3 | -0.034±0.962 | 0.007±0.980 |
| Component 4 | 0.009±0.987 | 0.012±0.976 |
| Component 5 | -0.017±0.992 | 0.028±1.022 |
| Component 6 | 0.040±0.986 | 0.016±0.992 |

**^a^** Difference was found between populations with and without gene data, p value <0.05.

**Table S3.** The principal components with their items of the absolute value of factor loading >0.3 in the homemade food frequency questionnaire

| **Principal components** | **Item information (** the absolute value of factor loading**)** |
| --- | --- |
| Component 1 | Rice (0.338), vegetables (0.333), red meat (0.329) |
| Component 2 | Smoked food (0.387), pickled food (0.391) |
| Component 3 | Wheat products (0.499), coarse grains (0.445) |
| Component 4 | Animal offal (0.316), blood products (0.352) |
| Component 5 | Formula milk (0.733), eggs (0.385) |
| Component 6 | Sugar (0.354), chocolate (0.315) |

**Table S4.** Age, body mass index (BMI) and BMI-z scores of children at each visit

| **Sample size** | **Overweight**  **/Obesity(%)** | **Mean ±Standard Deviation** | | |
| --- | --- | --- | --- | --- |
|  |  | **Age (month)** | **BMI (kg/m2)** | **BMI-Z score** |
| 2580 | 389(15.08) | 0 | 13.42±1.36 | -0.03±1.03 |
| 2507 | 376(15.00) | 3.09±0.30 | 17.32±1.52 | 0.41±0.96 |
| 2531 | 381(15.05) | 6.05±0.37 | 17.86±1.65 | 0.43±1.04 |
| 2447 | 369(15.08) | 9.08±0.32 | 17.73±1.51 | 0.47±0.97 |
| 2507 | 384(15.32) | 12.20±0.43 | 17.32±1.38 | 0.48±0.92 |
| 2496 | 372(14.90) | 18.19±0.41 | 16.41±1.20 | 0.32±0.86 |
| 2389 | 360(15.07) | 24.28±0.58 | 16.03±1.20 | 0.12±0.90 |
| 2327 | 347(14.91) | 30.41±0.81 | 15.83±1.29 | 0.08±0.97 |
| 2482 | 373(15.03) | 36.14±1.15 | 15.93±1.37 | 0.27±1.01 |
| 2161 | 323(14.95) | 48.64±1.01 | 15.95±1.74 | 0.38±1.12 |
| 1809 | 270(14.93) | 60.40±1.20 | 15.51±1.65 | 0.10±1.10 |
| 1492 | 221(14.81) | 72.44±1.48 | 16.03±2.10 | 0.34±1.25 |

**Table S5.** Association of SNPs with children’s BMIz scores and overweight/obese using a generalized estimating equation

| **Variables** | **Association of BMIz score**  **[β (95%CI)]** **^a^** | |  | **Association of overweight/obese**  **[OR (95%CI)] ^a^** | |
| --- | --- | --- | --- | --- | --- |
|  | **In girls** | **In boys** |  | **In girls** | **In boys** |
| rs11676272 |  |  |  |  |  |
| A/A | Reference | Reference |  | Reference | Reference |
| G/A | 0.125(-0.214,0.464) | 0.010(-0.223,0.243) |  | 1.107(0.842,1.456) | 0.954(0.739,1.231) |
| G/G | 0.087(-0.351,0.525) | 0.099(-0.191,0.389) |  | 0.951(0.669,1.352) | 1.088(0.801,1.478) |
| rs12041852 |  |  |  |  |  |
| A/A | Reference | Reference |  | Reference | Reference |
| G/A | **-1.419(-2.751,-0.087)** | -0.167(-0.818,0.483) |  | 1.228(0.556,2.715) | **2.814(1.185,6.684)** |
| G/G | -1.241(-2.545,0.062) | -0.069(-0.700,0.562) |  | 1.735(0.811,3.715) | **3.000(1.290,6.697)** |
| rs12429545 |  |  |  |  |  |
| G/G | Reference | Reference |  | Reference | Reference |
| G/A | -0.019(-0.338,0.299) | 0.135(-0.091,0.360) |  | 0.975(0.851,1.267) | 1.028(0.608,1.307) |
| A/A | 0.148(-0.388,0.685) | 0.090(-0.352,0.533) |  | 1.475(0.932,2.335) | 1.068(0.710,1.606) |
| rs13130484 |  |  |  |  |  |
| C/C | Reference | Reference |  | Reference | Reference |
| C/T | 0.016(-0.285,0.318) | 0.006(-0.209,0.221) |  | 1.140(0.885,1.467) | 1.069(0.845,1.353) |
| T/T | **0.498(-0.035,1.031)** | 0.286(-0.073,0.645) |  | **2.049(1.342,3.128)** | 1.115(0.758,1.640) |
| rs1421085 |  |  |  |  |  |
| T/T | Reference | Reference |  | Reference | Reference |
| C/T | -0.082(-0.446,0.282) | 0.026(-0.249,0.300) |  | 1.117(0.835,1.494) | 1.006(0.758,1.336) |
| C/C | -0.186(-2.198,1.827) | 0.403(-0.653,1.459) |  | 1.534(0.708,3.325) | 1.350(0.481,3.788) |
| rs3829849 |  |  |  |  |  |
| C/C | Reference | Reference |  | Reference | Reference |
| C/T | 0.113(-0.290,0.515) | 0.074(-0.188,0.336) |  | 1.087(0.770,1.536) | **0.759(0.579,0.996)** |
| T/T | **0.945(0.069,1.821)** | **-0.969(-1.343,-0.595)** |  | 2.162(0.648,7.207) | — |
| rs543874 |  |  |  |  |  |
| C/C | Reference | Reference |  | Reference | Reference |
| C/T | -0.186(-0.526,0.155) | -0.048(-0.272,0.177) |  | 1.185(0.907,1.548) | 0.817(0.635,1.051) |
| T/T | -0.269(-0.965,0.427) | -0.044(-0.628,0.540) |  | 1.306(0.830,2.055) | 1.087(0.599,1.973) |
| rs6567160 |  |  |  |  |  |
| A/A | Reference | Reference |  | Reference | Reference |
| G/A | 0.238(-0.654,1.129) | 0.044(-0.466,0.554) |  | 0.841(0.431,1.641) | 0.968(0.510,1.838) |
| G/G | 0.056(-0.825,0.938) | 0.012(-0.477,0.500) |  | 0.779(0.409,1.485) | 1.053(0.562,1.973) |
| rs7132908 |  |  |  |  |  |
| G/G | Reference | Reference |  | Reference | Reference |
| G/A | -0.048(-0.381,0.284) | 0.138(-0.082,0.359) |  | 1.016(0.789,1.308) | 0.905(0.717,1.141) |
| A/A | 0.217(-0.240,0.674) | 0.449(-0.015,0.913) |  | 1.375(0.885,2.137) | 0.747(0.363,1.538) |
| rs8092503 |  |  |  |  |  |
| A/A | Reference | Reference |  | Reference | Reference |
| G/A | -0.141(-0.491,0.208) | 0.109(-0.127,0.346) |  | 0.877(0.674,1.141) | 1.053(0.818,1.356) |
| G/G | -0.192(-0.620,0.236) | -0.057(-0.335,0.221) |  | 0.909(0.638,1.295) | 1.029(0.745,1.423) |
| rs987237 |  |  |  |  |  |
| A/A | Reference | Reference |  | Reference | Reference |
| G/A | -0.292(-0.635,0.051) | 0.044(-0.182,0.271) |  | 1.092(0.833,1.431) | 1.045(0.822,1.329) |
| G/G | -0.710(-1.444,0.024) | -0.294(-0.935,0.346) |  | 0.752(0.366,1.546) | 1.429(0.729,2.800) |

^a^ Adjustment for maternal age, education, parity, smoking and drinking during pregnancy and household income; Boldtype represents p values less than 0.05.

**Table S6.** Sensitivity analyses for association of the interaction of GMS risk and PRS risk with overweight/obese using a generalized estimating equation

| **GMS risk** | **PRS risk** | **Association of BMIz score** | |  | **Association of overweight/obese** | |
| --- | --- | --- | --- | --- | --- | --- |
| **In the population of Girls** | | **β (95%CI) ^a^**  **（N= 927）** | **β (95%CI) ^b^**  **（N= 1134）** |  | **OR (95%CI) ^a^**  **（N= 927）** | **OR (95%CI) ^b^**  **（N= 1134）** |
| Risk-free | Low-risk | 0(Reference) | 0(Reference) |  | 1.00(Reference) | 1.00(Reference) |
| Risk-free | High-risk | 0.702(-0.272,1.675) | 0.225(-0.260,0.709) |  | **2.699(1.591,4.577)** | **1.755(1.128,2.732)** |
| Risky | Low-risk | **0.823(0.119,1.527)** | **0.431(0.069,0.792)** |  | **2.864(1.791,4.579)** | **1.725(1.180,2.524)** |
| Risky | High-risk | 0.544(-0.350,1.439) | **0.462(0.027,0.896)** |  | **3.215(1.857,5.566)** | **2.092(1.371,3.192)** |
| Additive interaction: |  |  |  |  |  |  |
| AP(95%CI) |  | -0.913(-2.737,0.911) | -0.128(-1.321,1.064) |  | -0.419(-1.024,0.186) | -0.186(-0.648,0.277) |
| RERI(95%CI) |  | -1.572(-4.277,1.133) | -0.204(-2.086,1.678) |  | -1.347(-3.161,0.466) | -0.389(-1.340,0.562) |
| Synergy index(95%CI) |  | 0.315(0.045,2.203) | 0.742(0.066,8.390) |  | 0.622(0.346,1.118) | 0.737(0.374,1.454) |
| Multiplication interaction: |  |  |  |  |  |  |
| P for interaction |  | 0.136 | 0.084 |  | **<0.001** | **0.006** |
| **In the population of Boys** | | **β (95%CI) ^a^**  **（N= 976）** | **β (95%CI) ^b^**  **（N= 1139）** |  | **OR (95%CI) ^a^**  **（N= 976）** | **OR (95%CI) ^b^**  **（N= 1139）** |
| Risk-free | Low-risk | 0(Reference) | 0(Reference) |  | 1.00(Reference) | 1.00(Reference) |
| Risk-free | High-risk | 0.213(-0.230,0.657) | 0.052(-0.348,0.452) |  | 0.768(0.414,1.424) | 0.781(0.539,1.132) |
| Risky | Low-risk | 0.133(-0.178,0.445) | 0.041(-0.255,0.337) |  | **1.893(1.311,2.732)** | **1.291(1.013,1.646)** |
| Risky | High-risk | **0.561(0.176,0.945)** | 0.225(-0.129,0.579) |  | **2.399(1.556,3.699)** | **1.751(1.311,2.340)** |
| Additive interaction: |  |  |  |  |  |  |
| AP(95%CI) |  | 0.213(-0.178,0.604) | 0.125(-0.303,0.554) |  | 0.308(-0.026,0.642) | **0.388(0.144,0.632)** |
| RERI(95%CI) |  | 0.373(-0.349,1.094) | 0.157(-0.388,0.702) |  | 0.738(-0.167,1.643) | **0.679(0.196,1.163)** |
| Synergy index(95%CI) |  | 1.982(0.329,11.940) | 2.650(0.007,976.853) |  | 2.117(0.631,7.106) | 10.484(0.012,9088.858) |
| Multiplication interaction: |  |  |  |  |  |  |
| P for interaction |  | **0.030** | 0.613 |  | **<0.001** | **<0.001** |

^a^ Adjustment for maternal age, education, parity, smoking and drinking during pregnancy, household income, breastfeeding pattern and periods, physical activity and dietary pattern; ^b^ Excluded children with preterm birth and low and high birth weight; adjustment for maternal age, education, parity, smoking and drinking during pregnancy and household income. Boldtype representing a p value was less than 0.05.

**Table S7.** Association of SNP and PRS with the trajectory group of children’s BMIz scores

| **Variables** | **No. (%)** | **Girls** | | | | |  | **No. (%)** | **Boys** | | | | |
| --- | --- | --- | --- | --- | --- | --- | --- | --- | --- | --- | --- | --- | --- |
|  |  | **Medium BMIz trajectory** | |  | **High BMIz trajectory** | |  |  | **Medium BMIz trajectory** | |  | **High BMIz trajectory** | |
|  |  | ***OR(95%CI) ^a^*** |  |  | ***OR(95%CI) ^a^*** |  |  |  | ***OR(95%CI) ^a^*** |  |  | ***OR(95%CI) ^a^*** |  |
| rs11676272 |  |  |  |  |  |  |  |  |  |  |  |  |  |
| A/A | 242(32.97) | Reference |  |  | Reference |  |  | 256(34.22) | Reference |  |  | Reference |  |
| G/A | 357(48.64) | 1.326(0.914,1.924) |  |  | 1.403(0.818,2.406） |  |  | 359(48.99) | 0.686(0.457,1.030) |  |  | 0.776(0.481,1.251） |  |
| G/G | 135(13.39) | 0.975(0.605,1.572) |  |  | 1.354(0.698,2.627) |  |  | 133(17.78) | 1.177(0.671,2.064) |  |  | 1.147(0.595,2.209) |  |
| rs12041852 |  |  |  |  |  |  |  |  |  |  |  |  |  |
| A/A | 18(2.45) | Reference |  |  | Reference |  |  | 17(2.28) | Reference |  |  | Reference |  |
| G/A | 203(27.66) | 1.175(0.425,3.249) |  |  | 2.140(0.247,18.540) |  |  | 210(28.11) | 1.242(0.423,3.648) |  |  | 2.53×10^5^(<0.001,∞) |  |
| G/A | 513(69.89) | 1.502(0.557,4.054) |  |  | 4.158(0.498,34.690) |  |  | 520(69.61) | 1.326(0.465,3.780) |  |  | 3.14×10^5^(<0.001,∞) |  |
| rs12429545 |  |  |  |  |  |  |  |  |  |  |  |  |  |
| G/G | 421(57.51) | Reference |  |  | Reference |  |  | 424(56.91) | Reference |  |  | Reference |  |
| G/A | 258(35.25) | 0.906(0.635,1.292) |  |  | 0.845(0.508,1.404) |  |  | 269(36.11) | 0.800(0.547,1.170) |  |  | 0.771(0.490,1.212) |  |
| A/A | 53(7.24) | 1.039(0.535,2.020) |  |  | 1.007(0.401,2.532) |  |  | 52(6.98) | 1.457(0.658,3.225) |  |  | 1.443(0.586,3.556) |  |
| rs13130484 |  |  |  |  |  |  |  |  |  |  |  |  |  |
| C/C | 355(48.37) | Reference |  |  | Reference |  |  | 377(50.40) | Reference |  |  | Reference |  |
| C/T | 319(43.46) | 1.000(0.708,1.412) |  |  | 1.112(0.672,1.839) |  |  | 306(40.91) | 0.708(0.484,1.035) |  |  | 0.782(0.498,1.226) |  |
| T/T | 60(8.17) | 0.789(0.408,1.524) |  |  | **2.331(1.084,5.013)** |  |  | 65(8.69) | 0.811(0.408,1.615) |  |  | 1.183(0.547,2.559) |  |
| rs1421085 |  |  |  |  |  |  |  |  |  |  |  |  |  |
| T/T | 560(76.70) | Reference |  |  | Reference |  |  | 589(78.74) | Reference |  |  | Reference |  |
| C/T | 161(21.93) | 1.303(0.864,1.965) |  |  | 1.091(0.608,1.954) |  |  | 149(19.92) | **0.543(0.354,0.834)** |  |  | **0.539(0.320,0.907)** |  |
| C/C | 10(1.36) | 2.000(0.407,9.834) |  |  | 0.988(0.087,11.187) |  |  | 10(1.34) | 0.665(0.161,2.747) |  |  | 0.265(0.027,2.619) |  |
| rs3829849 |  |  |  |  |  |  |  |  |  |  |  |  |  |
| C/C | 622(84.74) | Reference |  |  | Reference |  |  | 619(82.75) | Reference |  |  | Reference |  |
| C/T | 107(14.58) | 1.024(0.637,1.646) |  |  | 1.139(0.587,2.212) |  |  | 128(17.11) | 0.750(0.473,1.188) |  |  | 0.167(0.382,1.181) |  |
| T/T | 5(0.68) | **—** |  |  | — |  |  | 1(0.13) | — |  |  | — |  |
| rs543874 |  |  |  |  |  |  |  |  |  |  |  |  |  |
| C/C | 479(65.26) | Reference |  |  | Reference |  |  | 467(62.43) | Reference |  |  | Reference |  |
| C/T | 222(30.25) | 0.999(0.691,1.443) |  |  | 1.317(0.797,2.175） |  |  | 250(33.42) | 1.271(0.860,1.880) |  |  | 0.835(0.520,1.339) |  |
| T/T | 33(4.50) | 1.799(0.751,4.308) |  |  | 0.962(0.240,3.857) |  |  | 31(4.14) | 0.777(0.320,1.888) |  |  | 0.861(0.316,2.344) |  |
| rs6567160 |  |  |  |  |  |  |  |  |  |  |  |  |  |
| A/A | 27(3.68) | Reference |  |  | Reference |  |  | 32(4.28) | Reference |  |  | Reference |  |
| G/A | 247(33.65) | 0.402(0.131,1.235) |  |  | 0.345(0.087,1.372) |  |  | 257(34.36) | 1.253(0.517,3.039) |  |  | 1.299(0.450,3.748) |  |
| G/A | 460(62.67) | 0.419(0.139,1.267) |  |  | 0.375(0.097,1.449) |  |  | 459(61.36) | 1.326(0.559,3.145) |  |  | 1.313(0.467,3.694) |  |
| rs7132908 |  |  |  |  |  |  |  |  |  |  |  |  |  |
| G/G | 421(57.36) | Reference |  |  | Reference |  |  | 441(58.96) | Reference |  |  | Reference |  |
| G/A | 268(36.51) | 0.870(0.614,1.234) |  |  | 0.958(0.883,1.039) |  |  | 275(36.76) | 0.998(0.678,1.468) |  |  | 1.142(0.728,1.792) |  |
| A/A | 45(6.13) | 1.069(0.516,2.217) |  |  | 0.879(0.574,1.448) |  |  | 32(4.28) | 0.647(0.282,1.487) |  |  | 0.501(0.163,1.538) |  |
| rs8092503 |  |  |  |  |  |  |  |  |  |  |  |  |  |
| A/A | 248(33.83) | Reference |  |  | Reference |  |  | 273(36.50) | Reference |  |  | Reference |  |
| G/A | 359(48.98) | 0.750(0.515,1.092) |  |  | 0.675(0.399,1.144) |  |  | 336(44.92) | 1.390(0.932,2.071) |  |  | 1.567(0.979,2.509) |  |
| G/G | 126(17.19) | 0.750(0.690,1.949) |  |  | 1.132(0.564,2.272) |  |  | 139(18.58) | **1.744(1.028,2.959)** |  |  | 1.403(0.739,2.661) |  |
| rs987237 |  |  |  |  |  |  |  |  |  |  |  |  |  |
| A/A | 528(71.93) | Reference |  |  | Reference |  |  | 531(70.99) | Reference |  |  | Reference |  |
| G/A | 194(26.43) | 1.194(0.813,1.753) |  |  | 1.060(0.615,1.828) |  |  | 198(26.47) | 0.742(0.498,1.104) |  |  | 0.977(0.917,1.041) |  |
| G/G | 12(1.63) | 2.928(0.623,13.758) |  |  | — |  |  | 19(2.54) | 1.679(0.464,6.073) |  |  | 0.628(0.387,1.019) |  |

The low BMI trajectory was referred to as the reference group. Adjustment for maternal age, education, parity, smoking and drinking during pregnancy, household income; bold type represents p values less than 0.05.

**Table S8.** Sensitivity analyses for association of the interaction of GMS risk and PRS risk with children’s BMIz score trajectory group

| **GMS risk** | **PRS risk** | **Medium BMIz trajectory** | |  | **High BMIz trajectory** | |
| --- | --- | --- | --- | --- | --- | --- |
| **In the population of Girls** |  | OR((95%CI) ^a^  (N = 277) | OR((95%CI) **^b^**  (N = 370) |  | OR((95%CI) ^a^  (N = 79) | OR((95%CI) **^b^**  (N = 91) |
| Risk-free | Low-risk | 1.00(Reference) | 1.00(Reference) |  | 1.00(Reference) | 1.00(Reference) |
| Risk-free | High-risk | 2.076(0.967,4.456) | 1.385(0.736,2.606) |  | **5.207(2.784,9.737)** | **2.038(1.232,3.372)** |
| Risky | Low-risk | 1.603(0.937,2.744) | 1.444(0.940,2.220) |  | **5.207(3.436,7.891)** | **1.744(1.249,2.435)** |
| Risky | High-risk | 1.845(0.961,3.543) | 1.614(0.941,2.768) |  | **7.508(4.470,12.611)** | **2.787(1.806,4.301)** |
| Additive interaction: |  |  |  |  |  |  |
| AP(95%CI) |  | -0.451(-1.508,0.606) | -0.134(-0.860,0.593) |  | -0.254(-0.894,0.387) | 0.002(-0.496,0.500) |
| RERI(95%CI) |  | -0.832(-2.666,1.001) | -0.216(-1.357,0.926) |  | -1.906(-6.251,2.440) | 0.005(-1.384,1.395) |
| Synergy index(95%CI) |  | 0.504(0.131,1.935) | 0.740(0.165,3.324) |  | 0.774(0.432,1.384) | 1.003(0.460,2.186) |
| Multiplication interaction: |  |  |  |  |  |  |
| P for interaction |  | 0.077 | 0.060 |  | **<0.001** | **0.016** |
| **In the population of Boys** |  | OR((95%CI) ^a^  (N = 265) | OR((95%CI) **^b^**  (N = 355) |  | OR((95%CI) ^a^  (N = 121) | OR((95%CI) **^b^**  (N = 130) |
| Risk-free | Low-risk | 1.00(Reference) | 1.00(Reference) |  | 1.00(Reference) | 1.00(Reference) |
| Risk-free | High-risk | 0.485(0.206,1.145) | 0.495(0.240,1.022) |  | 0.661(0.325,1.344) | 0.643(0.264,1.567) |
| Risky | Low-risk | **0.509(0.277,0.937)** | **0.553(0.333,0.918)** |  | 0.901(0.535,1.519) | 0.732(0.393,1.363) |
| Risky | High-risk | 0.633(0.305,1.314) | 0.819(0.445,1.507) |  | 1.208(0.652,2.238) | 1.031(0.494,2.151) |
| Additive interaction: |  |  |  |  |  |  |
| AP(95%CI) |  | **1.009(0.006,2.011)** | **0.941(0.286,1.596)** |  | 0.535(-0.022,1.091) | **0.636(0.128,1.143)** |
| RERI(95%CI) |  | **0.638(0.103,1.174)** | **0.770(0.270,1.271)** |  | 0.646(-0.052,1.343) | **0.655(0.128,1.143)** |
| Synergy index(95%CI) |  | **0.365(0.128,1.040)** | 0.190(0.016,2.287) |  | -0.475(—,—) | -0.049(—,—) |
| Multiplication interaction: |  |  |  |  |  |  |
| P for interaction |  | 0.844 | 0.302 |  | 0.614 | 0.952 |

Abbreviation; AP, the attributable proportion due to interaction; REMI: the relative excess risk due to interaction; Normative BMI trajectory was referred to as the reference group; ^a^ Adjustment for maternal age, education, parity, smoking and drinking during pregnancy, household income, breastfeeding pattern and periods, physical activity and dietary pattern; ^b^ Excluded children with preterm birth and low and high birth weight; adjustment for maternal age, education, parity, smoking and drinking during pregnancy and household income. Boldtype representing a p value was less than 0.05.

**Table S9**. Effects of a new polygenic risk score (PRS_β_) based on these SNPs according to a weighted method for each SNP and its interaction with gestational metabolic syndrome (GMS) risk on children’s body mass index (BMI) z-score trajectory latents

| **Variables** | **Girls** | | | | |  | **Boys** | | | | |
| --- | --- | --- | --- | --- | --- | --- | --- | --- | --- | --- | --- |
|  | **Medium BMIz trajectory** | |  | **High BMIz trajectory** | |  | **Medium BMIz trajectory** | |  | **High BMIz trajectory** | |
|  | ***OR(95%CI) ^a^***  **(N = 412)** |  |  | ***OR(95%CI) ^a^***  **(N = 106)** |  |  | ***OR(95%CI) ^a^***  **(N = 403)** |  |  | ***OR(95%CI) ^a^***  **(N = 168)** |  |
| PRS_β_ | 1.906(0.463,7.850) |  |  | 6.068(0.839,43.894) |  |  | 0.641(0.143,2.883) |  |  | 0.430(0.072,2.556) |  |
| Low risk (PRS_β_ <P_75_) | Reference |  |  | Reference |  |  | Reference |  |  | Reference |  |
| High risk (PRS_β_ >P_75_) | 1.321(0.882,1.978) |  |  | **2.094(1.237,3.545)** |  |  | 1.019(0.671,1.5480 |  |  | 1.024(0.626,1.675) |  |
| Interaction between GMS and PRS_β_ |  |  |  |  |  |  |  |  |  |  |  |
| GMS no risk and PRS_β_ low risk | Reference |  |  | Reference |  |  | Reference |  |  | Reference |  |
| GMS no risk and PRS_β_ high risk | **2.115(1.058,4.229)** |  |  | **2.599(1.458,4.633)** |  |  | 0.701(0.337,1.460) |  |  | 0.873(0.470,1.622) |  |
| GMS low-to-high risk and PRS_β_ low risk | **1.624(1.092,2.415)** |  |  | **1.998(1.465,2.723)** |  |  | 0.614(0.385,0.981) |  |  | 0.950(0.636,1.420) |  |
| GMS low-to-high risk and PRS_β_ high risk | 1.675(0.969,2.896) |  |  | **3.714(2.396,5.756)** |  |  | 0.743(0.409,1.351) |  |  | 1.046(0.627,1.744) |  |
| Additive interaction: |  |  |  |  |  |  |  |  |  |  |  |
| AP(95%CI) | -0.635(-1.738,0.469) |  |  | 0.032(-0.495,0.558) |  |  | 0.575(-0.261,1.411) |  |  | 0.213(-0.436,0.862) |  |
| RERI(95%CI) | -1.064(-2.743,0.615) |  |  | 0.117(-1.863,2.097) |  |  | 0.427(-0.183,1.038) |  |  | 0.223(-0.466,0.912) |  |
| Synergy index(95%CI) | 0.388(0.100,1.505) |  |  | 1.045(0.494,2.210) |  |  | 0.375(0.083,1.701) |  |  | -0.260(—,—) |  |
| Multiplication interaction: |  |  |  |  |  |  |  |  |  |  |  |
| P for interaction | **0.028** |  |  | **0.001** |  |  | 0.133 |  |  | 0.932 |  |

^a^ Adjustment for maternal age, education, parity, smoking and drinking during pregnancy, household income; Boldtype representing a p value was less than 0.05.

**
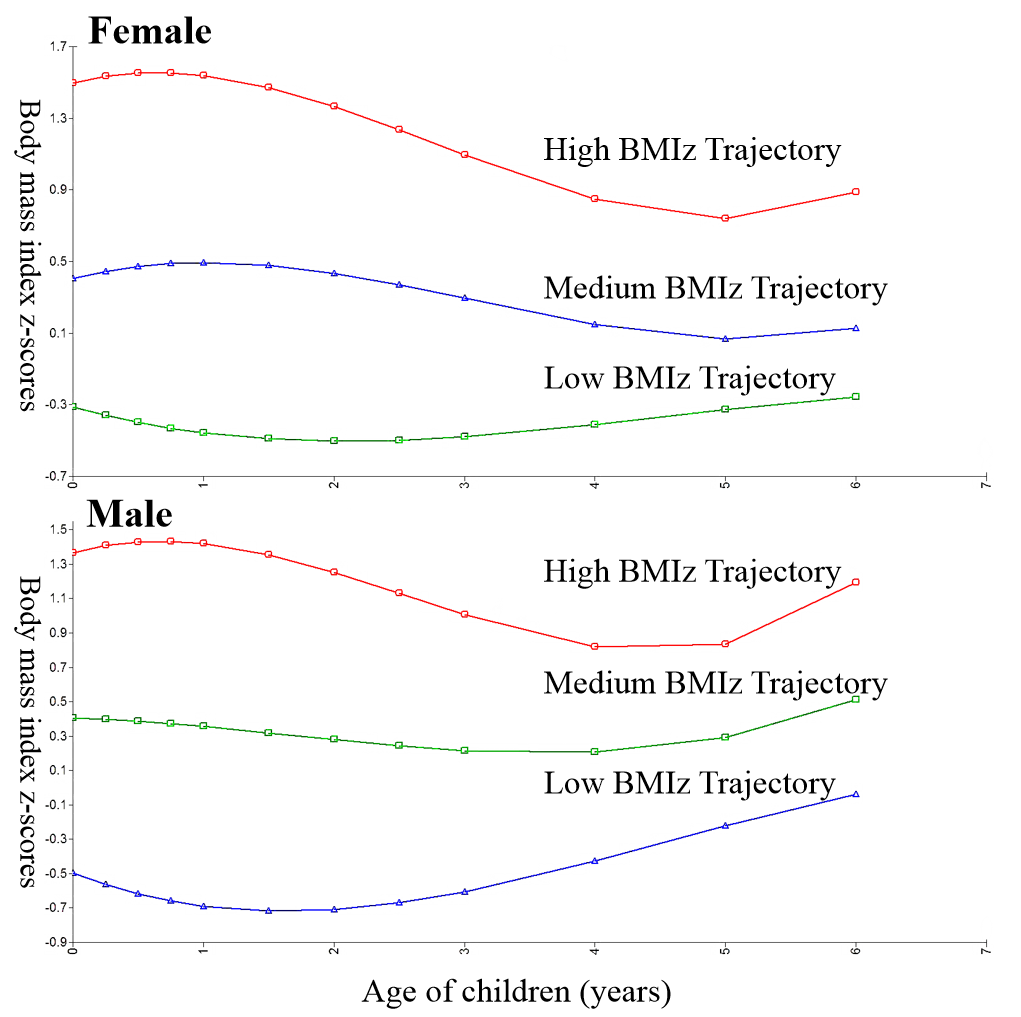
**

**Figure S1.** Horizontal curve of the sample mean based on three classes of latent class growth model (LCGM) in body mass index for age z-scores in girls (N = 1 276) and boys (N = 1 327)


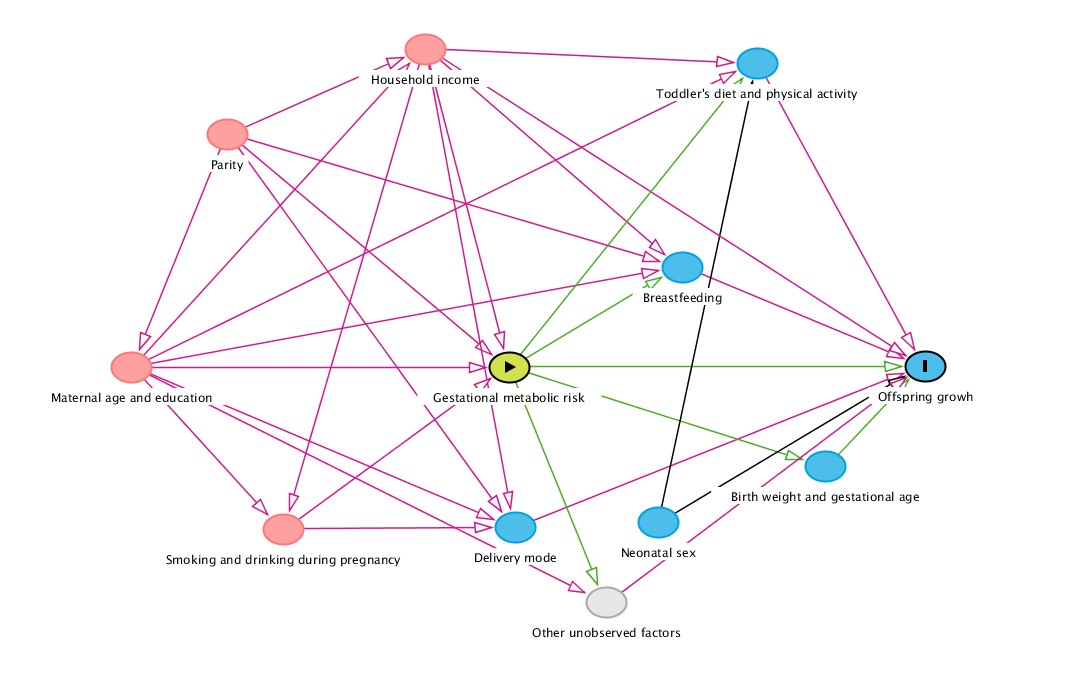


**Figure S2**. The directed acyclic graph (DAG) for all potential confounders considered in the statistical analyses of Ma’anshan Birth Cohort, Anhui, China, recruited from May 2013 to September 2014. The directed paths from gestational metabolic risk to offspring growth represents the potential causal effect of interests that the present study aim to investigate. Other unobserved factors should be evaluated for their potential impacts in future studies.


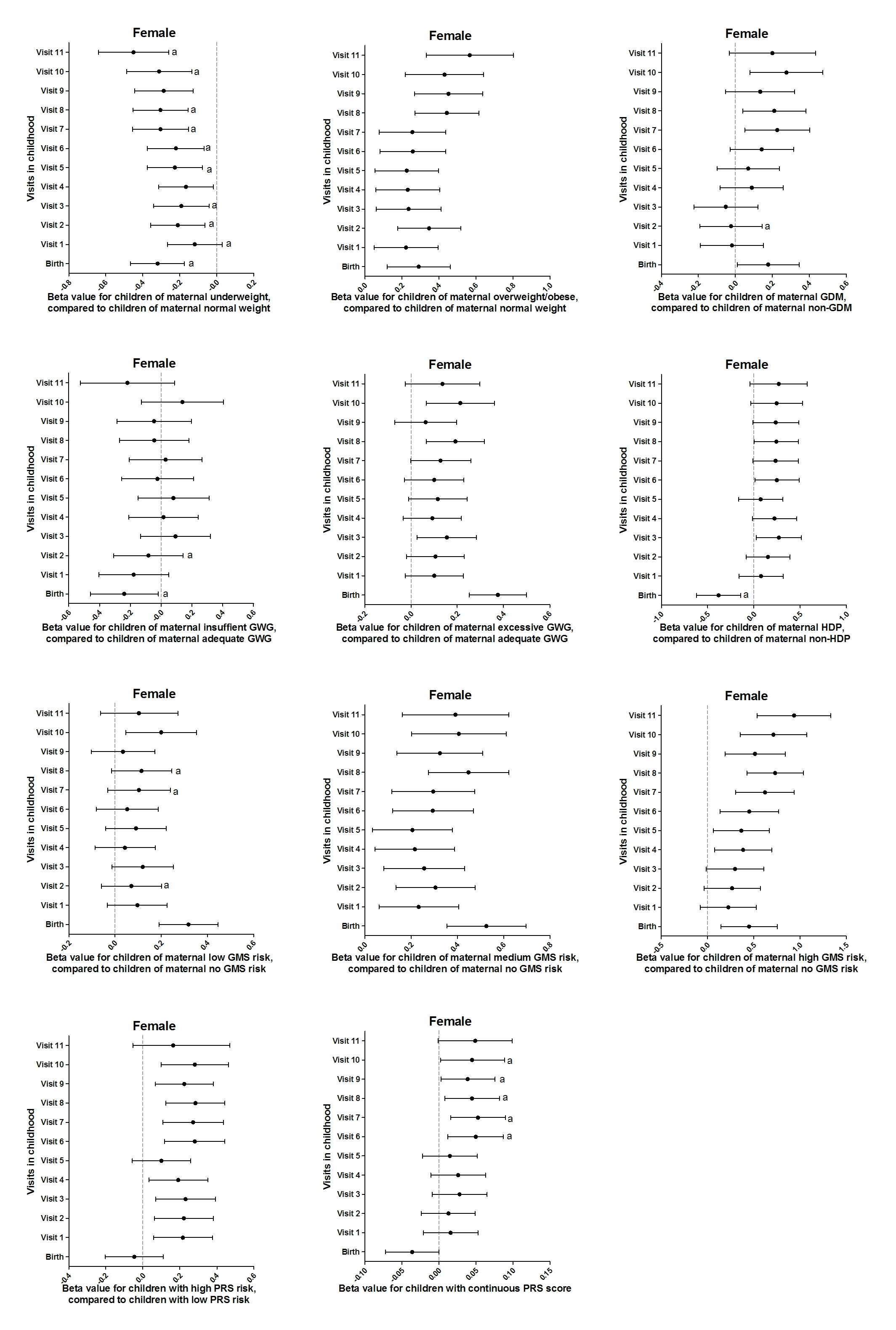


**Figure S3.** Association of gestational metabolic syndrome (GMS) parameters and polygenic risk scores (PRS) with girl’s body mass index z-scores at each visit (GWG, gestational weight gain; adjustment for maternal age, education, parity, smoking and drinking during pregnancy and household income; ^a^ a *p*-value for interaction with age <0.05).


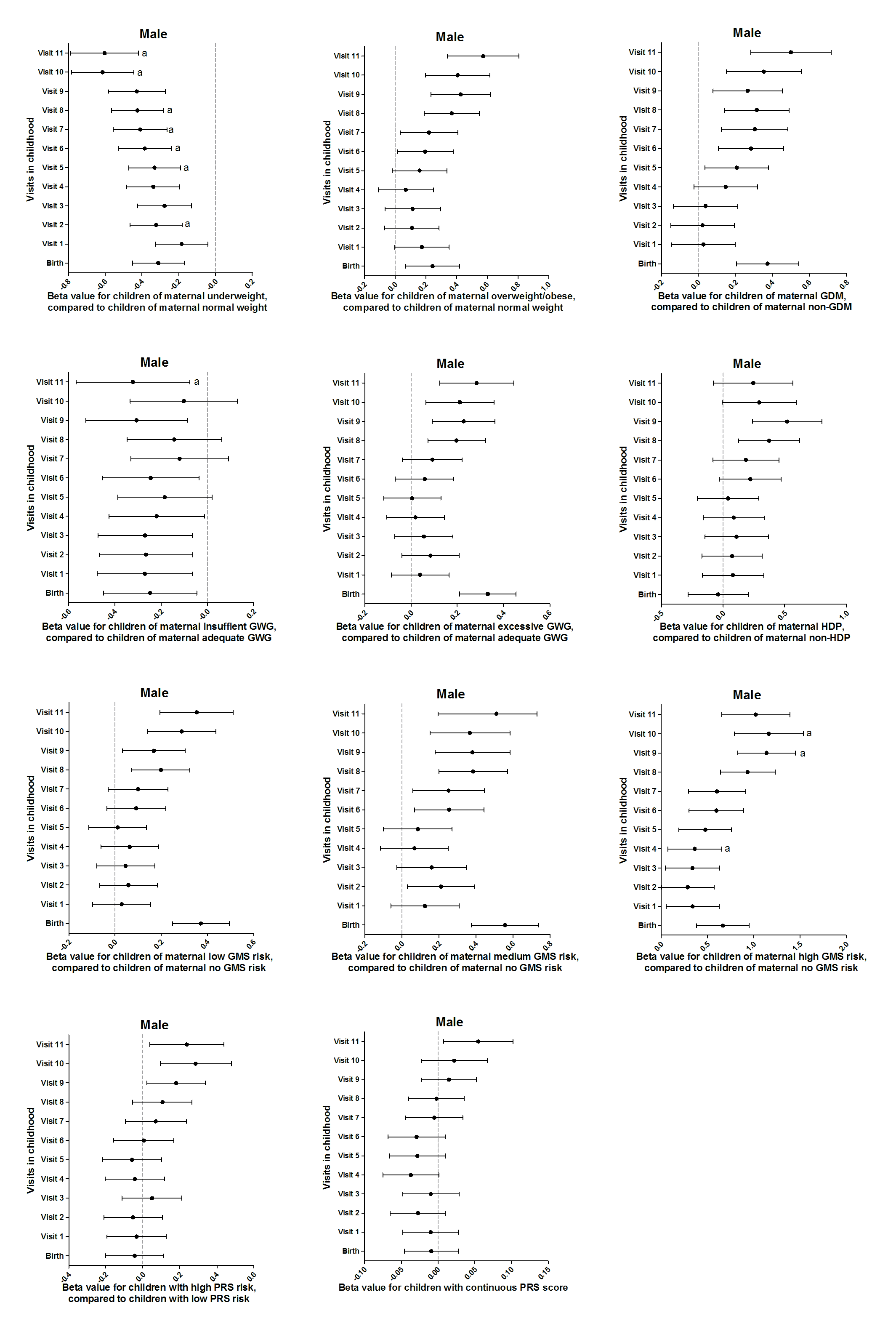


**Figure S4.** Association of gestational metabolic syndrome (GMS) parameters and polygenic risk scores (PRS) with boy’s body mass index z-scores at each visit (GWG, gestational weight gain; adjustment for maternal age, education, parity, smoking and drinking during pregnancy and household income; ^a^ a *p*-value for interaction with age <0.05).


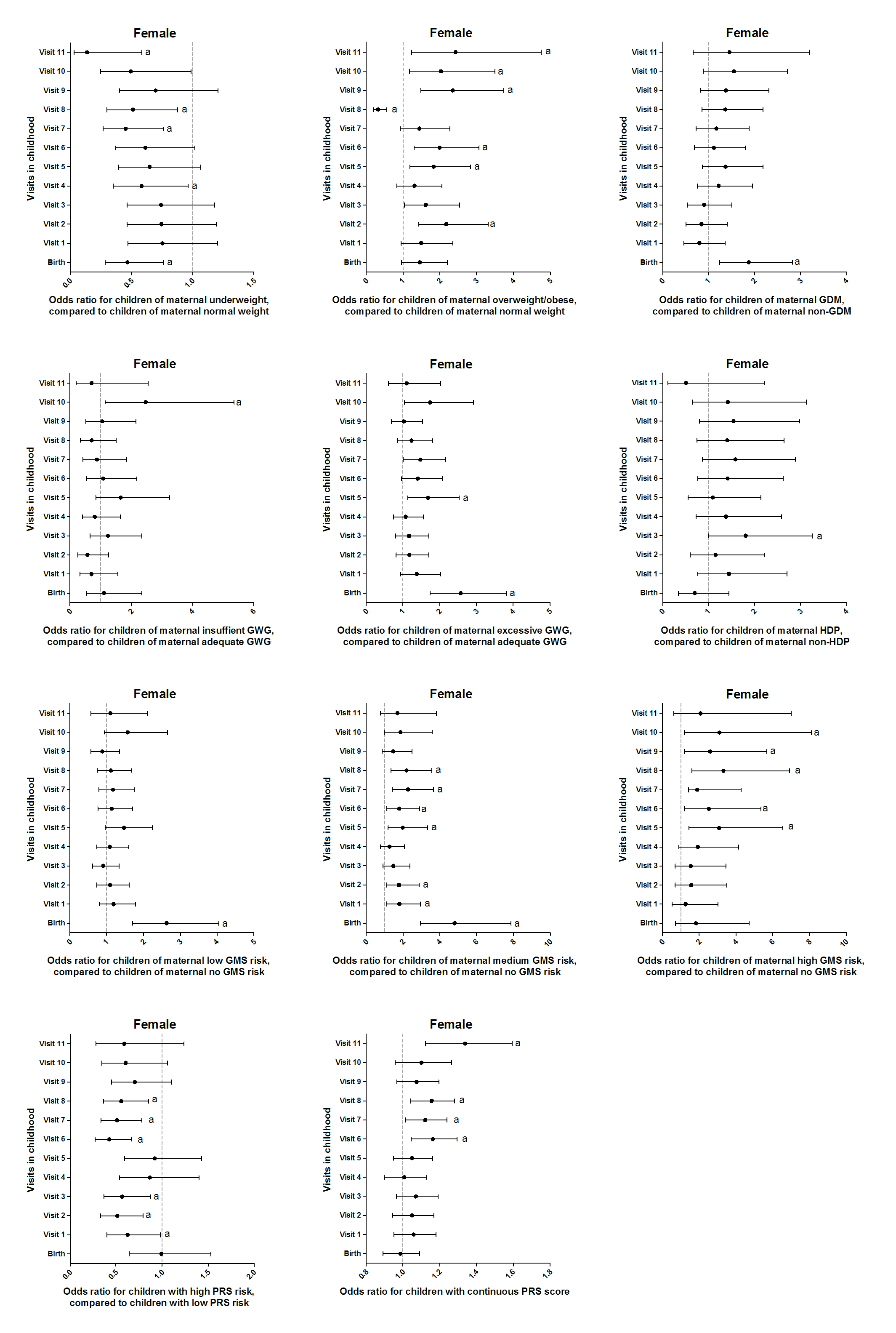


**Figure S5**. Association of gestational metabolic syndrome (GMS) parameters and polygenic risk scores (PRS) with girl’s overweight/obese risks at each visit (GWG, gestational weight gain; adjustment for maternal age, education, parity, smoking and drinking during pregnancy and household income; ^a^ a *p*-value for interaction with age <0.05).


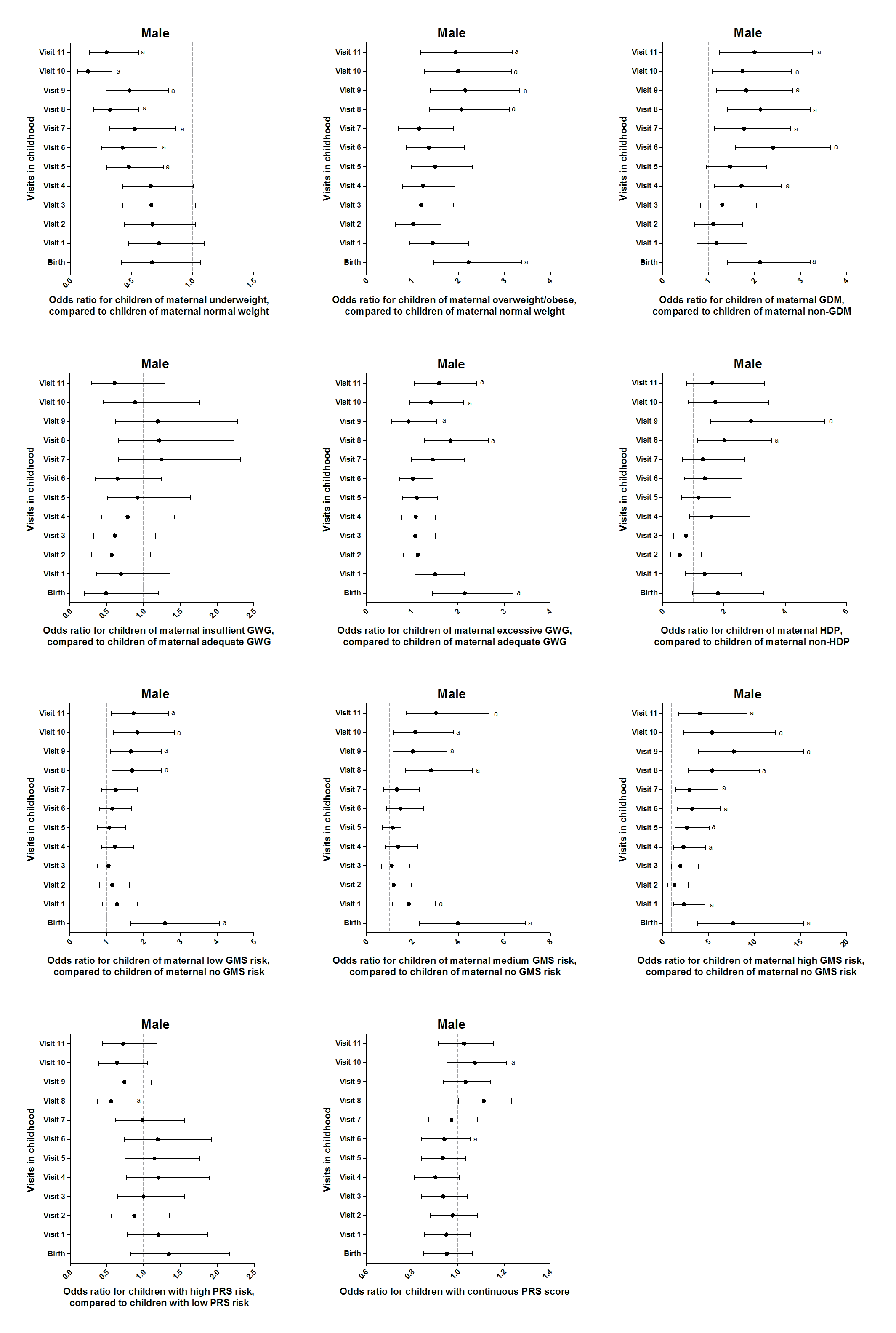


**Figure S6.** Association of gestational metabolic syndrome (GMS) parameters and polygenic risk scores (PRS) with boy’s overweight/obese risks at each visit (GWG, gestational weight gain; adjustment for maternal age, education, parity, smoking and drinking during pregnancy and household income; ^a^ a *p*-value for interaction with age <0.05).
